# Uncertainty-guided informative path planning for ecological monitoring using autonomous surface vehicles under Dubins motion constraints

**DOI:** 10.3389/frobt.2026.1823324

**Published:** 2026-07-21

**Authors:** Jalil Chavez-Galaviz, Meredith Bloss, Nina Mahmoudian

**Affiliations:** 1 School of Mechanical Engineering, Purdue University, West Lafayette, IN, United States; 2 Centre for Underwater Robotics Research (CIRS), University of Girona, Girona, Spain

**Keywords:** adaptive sampling, autonomous surface vessel, ecological monitoring, Gaussian process regression, informative path planning, marine mapping

## Abstract

Autonomous surface vehicles (ASVs) enable efficient in-situ data collection for large-scale ecological monitoring; however, effective environmental mapping requires planning strategies that account for not only informative measurements, but also vehicle motion constraints and limited mission resources. Existing approaches often rely on stationary environmental models or loosely coupled planning frameworks that do not fully exploit model uncertainty when generating feasible trajectories. To address these limitations, we propose a closed-loop informative path planning (IPP) framework that tightly integrates environmental modeling and trajectory generation for autonomous sampling. The proposed approach combines a nonstationary uncertainty representation using Gaussian Process Regression with Attentive Kernels (AK-GPR), uncertainty- guided adaptive sampling, and a Dubins-constrained RRT* planner to generate dynamically feasible and information-rich trajectories. The proposed framework is evaluated through staged experiments, including simulation and field validation, across representative ecological monitoring scenarios such as algal plume tracking, bathymetric mapping, and seagrass probability estimation. The results demonstrate that the proposed planner shows improved performance relative to the baseline planners in different initialization grid densities, with particularly strong performance in scenarios with limited prior information. In all environments, the proposed IPP framework achieved an average reduction of approximately 24% in mean absolute error (MAE) and 32% in predictive uncertainty compared to baseline planners, with improvements reaching up to 33% in MAE and 42%, respectively, in sparse initialization settings. These results demonstrate the benefits of a tightly coupled framework that balances uncertainty reduction and spatial coverage, enabling more efficient environmental exploration under realistic vehicle constraints.

## 1 Introduction

As a subset of the larger field of environmental robotics, marine robotics applies autonomous technologies to nautical domains as a supplement to traditional monitoring methods. The combination of increased interest, rapid technological advancement, and potential for impact suggests that marine robotics is a promising field for innovation by improving the amount and quality of data that can be collected to assess marine health. The rise in activity within marine monitoring and, more specifically, within marine robotics can be partially attributed to Blue Economy initiatives (United Nations Conference on Trade and Development UNCTAD, 2014) that link ocean sustainability and economic growth.

The traditional manual monitoring schema employed in marine monitoring spans a variety of methods, including hand-sampling techniques. The specific method used and the frequency and quality of the resultant measurement vary depending on the site accessibility, staffing, and financial resources (Behmel et al., 2016). The resultant lack of high-quality usable data makes water monitoring difficult, either due to inadequate sampling technologies, difficulties with manpower and funding, or differing sampling standards. Semi-autonomous monitoring techniques, such as drifters and stationary sensors as in (Meyerjürgens et al., 2019; Lumpkin et al., 2017; Panagopoulos et al., 2021), can help reduce the sampling burden. Fully autonomous solutions can further improve this process by enabling more focused sampling.

One such application of marine monitoring is plume detection. Plumes span a variety of compositions, including thermal (Cannell et al., 2006), salt-freshwater tidal (Horner-Devine et al., 2015), oil (Hwang et al., 2020), and algal plumes (Fonseca et al., 2021). One specific type of plume of interest is that of algal plumes. These large algae growths can be found in both freshwater and saltwater bodies and can cause fish and shellfish contamination (Hallegraeff, 2003), toxin presence, and oxygen depletion (Anderson, 2009; Hallegraeff, 2003).

However, not all forms of marine monitoring and mapping focus on plumes. Another application of monitoring technology is occupancy and probability mapping. This can include archaeological mapping on the seafloor (e.g., Ravnås et al., 2023) and natural artifact mapping, as with seagrass (e.g., Guerrero-Font et al., 2021; Grimaldi et al., 2025). Seagrass mapping is useful as an indicator of ocean health. Underwater seagrass meadows, such as *Posidonia oceanica* meadows in the Mediterranean, store carbon dioxide and can help improve water quality (Baiocchi et al., 2024). Monitoring the presence and changes in these meadows can act as an indicator of adverse ecological changes and overall ocean health (Marbà et al., 2014).

In both of these environmental applications, algorithms that enable more efficient use of limited resources while systematically reducing environmental uncertainty are particularly relevant for ecological decision-making and ecosystem restoration efforts. This may include practices such as seagrass replanting, habitat protection zoning, and algal bloom mitigation. These interventions often involve substantial manual labor, specialized technical expertise, and high financial costs, which can limit their scalability (Unsworth and Rees, 2025). Adaptive sampling techniques, where the sampling strategy is updated as data is collected, can help improve data collection efficiency and quality. Autonomous robotic platforms capable of strategically collecting information to reduce predictive uncertainty and operational costs (Berget et al., 2018) can support managers in identifying priority intervention areas earlier, assessing habitat degradation with greater confidence and allocating restoration resources more effectively.

Current adaptive sampling techniques can be divided into two categories: gradient-free and gradient-based methods. The efficacy of each method depends on the specific plume type or application. Some plumes, such as saline plumes, can be successfully tracked with gradient-based methods (Belkin et al., 2018). One such gradient-based method is level curve tracking (Fahad et al., 2018; Fahad et al., 2017a; Fahad et al., 2017b; Wang et al., 2019). However, gradient-based methods are sensitive to discontinuity in the plume shape, making them a poor choice for tracking discontinuous and patchy plumes, which are common (Hwang et al., 2020; Fahad et al., 2017a). In addition, many of these plumes are measured using point sensors; these cannot give the concentration gradient and divergence directly. Finally, for large-scale plumes, less dramatic changes in plume concentration may make it difficult to adequately track level curves.

In cases where a gradient-based method is not appropriate, a gradient-free method may be used. Typically, this consists of a combination of Bayesian Experimental Design (BED), Informative Path Planning (IPP), and Gaussian Process Regression (GPR) (Feng et al., 2023; Ma et al., 2017; Chen et al., 2022a; Mo-Bjørkelund et al., 2024; Rainforth et al., 2024). Such approaches are not limited to marine surveying; these approaches are visible in similar fields like geostatistics (Li and Deutsch, 2010). Generally, the goal of these approaches is to maximize information gain. This is done by identifying which prospective sample points can provide the most information about a given area. This sampling approach is coupled with a model, typically achieved with GPR, to drive exploration in tandem with the model. This coupled approach increases the relative information provided by each point in comparison to more traditional methods like frontier-based exploration (Rasmussen, 2004).

Recent informative path planning methods have also considered explicit uncertainty guarantees through coverage-based strategies such as HEXCover (Dutta et al., 2022) and GreedyCover (Jakkala et al., 2026). These approaches search for sensing locations that satisfy prescribed uncertainty bounds over a discretized space. They provide strong theoretical reconstruction guarantees, but typically rely on simplified Euclidean travel models, predefined candidate sensing sets, or geometric assumptions such as convex domains. Such formulations can be restrictive for marine robotic platforms that must satisfy constrained motion, obstacle avoidance, and finite mission budgets during online operation.

To improve sampling efficiency and model accuracy within the context of marine sampling, this paper proposes an informative path planning framework that integrates an Attentive-Kernel GPR (AK-GPR) with a Dubins-constrained ${\text{RRT}}^{*}$ planner to map marine environments. The objective is to develop a vehicle- and sensor-agnostic informative planning framework that tightly couples path planning and environmental modeling in a closed loop. Using the predictive uncertainty of the AK-GPR model to guide sampling decisions under realistic Dubins motion constraints, the proposed approach improves data efficiency while enhancing the fidelity of reconstructed maps of marine phenomena. Beyond mapping performance, the framework provides spatial information that can be applied directly for the ecological monitoring, assessment, and adaptive management of marine system.

To enable efficient and scalable environmental mapping with autonomous vehicles, we propose a unified framework that integrates learning and planning in a closed loop, with the following contributions.

- Trajectory-level informative path planning under Dubins constraints: We propose a continuous-space IPP framework that optimizes trajectory-level information gain under Dubins constraints, generating dynamically feasible paths that balance exploration and exploitation.
- Uncertainty-driven sampling through predictive variance guidance: We introduce an adaptive sampling strategy where AK—GPR predictive variance directly shapes the RRT* sampling distribution, enabling information-driven exploration through uncertainty-guided adaptive sampling.
- Closed-loop integration of learning and planning for resource-constrained missions: We develop a tightly coupled framework integrating AK—GPR modeling, adaptive sampling, and motion planning while explicitly accounting for budget constraints and online model updates.
- Comprehensive validation across simulation and field deployment: We validate the framework through extensive simulation and real-world experiments, including deployment on a WAM-V platform across multiple ecological mapping tasks.

The paper begins with a review of related work in IPP in Section 2. In Section 3, the informative motion planning problem is defined. This is followed by a discussion of Dubins-constrained ${\text{RRT}}^{*}$ and uncertainty modeling through the AK-GPR in Section 4. In Section 5, the algorithm is tested and validated by (1) baseline field testing of the AK-GPR algorithm, (2) deployment of the Dubins– ${\text{RRT}}^{*}$ algorithm in two obstacle-free applications, and deployment in (3) obstacle-rich environments. Finally, the conclusions and future work are presented in Section 6.

## 2 Related work

The use of Informative Path Planning (IPP) has become a leading strategy for autonomous vehicles regarding environmental mapping, where the objective is not to uniformly cover an area but to prioritize measurements that most improve the predictive model of the environment. Unlike classic mapping approaches, such as lawnmower or grid-based surveying (Cabreira et al., 2019), IPP prioritizes regions that are expected to reduce the model’s uncertainty, improving the predictive model. This makes IPP particularly valuable in environments where certain areas provide more meaningful information than others and when the vehicle operates under strict constraints on time, distance, or energy. Previous work shows that IPP is capable of maximizing the information gained while respecting a fixed budget, differentiating it from traditional shortest-path planning methods (Moon et al., 2022).

Early approaches to robotic exploration and navigation primarily focused on reaching predefined targets or minimizing geometric cost functions, often relying on graph-based search algorithms, heuristic optimization or predefined waypoint sequences (Tan et al., 2021). While these methods are effective for tasks such as shortest-path planning or goal-oriented navigation, they are less suitable for exploratory scenarios in which the objective is to adaptively reduce uncertainty in an unknown environment (Hollinger and Sukhatme, 2014; Bai et al., 2021). In such cases, uniformly visiting predefined locations may lead to inefficient use of limited resources, particularly when the information content of the environment is unevenly distributed.

As a way to improve efficiency, several works formulate environmental monitoring as a routing or scheduling problem. In these cases, the workspace is discretized into predefined sampling locations associated with fixed rewards or information metrics, and single or multi-robot trajectories are optimized to maximize coverage or accumulated utility under travel constraints (Mansfield et al., 2021; McCammon and Hollinger, 2021). These approaches are often posed as variants of the Team Orienteering Problem or mixed-integer routing formulations, enabling efficient task allocation and coordination under budget limitations. While effective for coverage-driven missions and scenarios with predefined task utilities, such methods typically operate over static reward maps and do not explicitly maintain a continuous probabilistic representation of the underlying environmental field. Consequently, sampling decisions are not directly informed by posterior uncertainty updates during execution.

In comparison, the use of GPR employed in most IPP systems (Jakkala and Akella, 2024) enables uncertainty-informed sampling that is updated during execution. The GP models the spatial field that is being mapped and provides both predictions and uncertainty estimates from taken measurements. This uncertainty acts as the feedback signal that guides the vehicle to locations that are expected to have the greatest information. While standard stationary kernels are commonly used, they may struggle in applications where there are different levels of variation across space, leading to over- or under-smoothing of certain areas. This reduces the accuracy of the GP model and affects planning performance. Recent work has shown that more accurate GP uncertainty estimates can significantly improve planning performance by enabling more targeted and intelligent sampling strategies. Quindeln et al. demonstrated this by constructing GP models from a small training set and applying an active learning procedure that iteratively selects the most informative samples (Quindlen and How, 2017). Guided by these findings, this work employs a GP model that is more flexible regarding the environment’s variability through an Attentive Kernel design (Chen et al., 2022b), while keeping the emphasis on how IPP uses these models to achieve efficient and adaptive mapping.

Alternative formulations of informative path planning emphasize explicit uncertainty guarantees rather than direct reward maximization. For instance, Jakkala et al. seeks the shortest path whose measurements ensure GP posterior uncertainty over a discretized domain remains below a user-defined threshold, using candidate sensing locations, evaluation points, and binary coverage relationships with approximation guarantees (Jakkala et al., 2026). Similarly, the HEXCover algorithm as introduced in Dutta et al. computes the minimum sensing set and shortest route that satisfy a prescribed estimation-error bound, assuming stationary Gaussian fields, convex domains, and Euclidean travel through geometric hexagonal tiling (Dutta et al., 2022). These assumptions may be restrictive within the context of marine robotics.

Other approaches to IPP rely on discrete graph-based search algorithms and greedy optimization heuristics, but these methods scale poorly in large continuous environments and cannot easily incorporate realistic vehicle motion constraints. To address these limitations, Hollinger and Sukhatme introduce sampling-based informative planners, specifically the rapidly exploring information gathering algorithms (RIG), which extend classical motion planners (iRRT, ${\text{RRT}}^{*}$ , RRG) to maximize information gathering while respecting a budget constraint (Hollinger and Sukhatme, 2014). The authors demonstrated that generating trajectories directly in continuous space leads to more accurate maps and lower GP uncertainty, compared to methods that rely on discrete branch-and-bound searches. In addition, they validated this on an autonomous surface vehicle (ASV) performing a wireless signal strength mapping, demonstrating the practical advantages of sampling-based IPP methods.

Recent work has explored model-based deployment scheduling for persistent monitoring, where GP models are used to predict the evolution of spatio-temporal phenomena and trigger robot deployments when predictive uncertainty exceeds a desired threshold (Masaba et al., 2024). In these formulations, the primary objective is to determine when to deploy robots and which discrete hotspot locations to sample to maintain accuracy while minimizing operational cost. Although GPs are used to model the environment and quantify uncertainty, the spatial domain is typically discretized into predefined candidate locations, and trajectory generation is handled separately using standard routing or coverage planners.

Further advances have incorporated adaptive replanning to continuously update the vehicle’s path as new measurements are collected. Hitz et al. proposed a continuous space IPP method combined with an evolutionary optimization and real time replanning, which allows an ASV to prioritize sampling in regions of high significance while maintaining enough exploration (Hitz et al., 2017). In field experiments, this approach captured complex environmental structures more efficiently than uniform sampling, reinforcing the value of uncertainty aware navigation in natural environments.

Additional work in marine robotics has emphasized the relationship between IPP and other adaptive sampling strategies. Reviews of informative planning for underwater and surface vehicles describe how computational complexity increases with environmental scale. To maintain computational feasibility in these instances, sampling-based motion planners, probabilistic roadmaps, or hybrid optimization techniques are strong candidates for adoption (Yu et al., 2024). Together, these contributions demonstrate that IPP provides an effective strategy for selecting sampling locations that meaningfully improve the field representation while satisfying practical vehicle motion and budget constraints.

Overall, the existing literature establishes IPP as a powerful methodology for continuous environmental mapping, with Gaussian Processes providing the probabilistic foundation for representing uncertainty. The integration of the GP modeling with sampling-based trajectory generation and adaptive replanning forms a strong framework for modern applications. However, many existing approaches rely on stationary covariance models, discrete hotspot selection, or loosely coupled motion planning frameworks that do not explicitly incorporate vehicle kinematic and resource constraints within the information-driven objective.

Unlike prior IPP approaches that (i) decouple environmental modeling from trajectory generation, (ii) rely on stationary kernel assumptions, or (iii) assume simplified Euclidean motion, this work introduces a closed-loop framework that tightly integrates nonstationary uncertainty modeling with trajectory-level planning under realistic Dubins motion constraints.

This work proposes a trajectory-based informative path planning framework that explicitly incorporates vehicle kinematic constraints while maximizing information gain and spatial coverage along feasible Dubins trajectories. By tightly coupling uncertainty modeling with a Dubins-constrained RRT* planner, the proposed approach balances exploration and exploitation, enabling more efficient mapping of heterogeneous environmental phenomena. This capability is particularly important for applications such as monitoring biological indicators, water quality parameters, and other ecological patterns using autonomous vehicles.

## 3 Problem statement

We consider an ASV tasked with learning an unknown spatially varying environmental field $f_{env}:R^{D}\to R$ defined over a D -dimensional workspace. As the vehicle traverses the environment, it acquires noisy observations $y=f_{env}\left(x\right)+\epsilon$ where $x\in R^{D}$ denotes the sampling location and $\epsilon ∼N\left(0,\sigma^{2}\right)$ represents the measurement noise. The vehicle configuration is defined as s=(x,y,ψ) , where x and y denote the planar position and ψ represents the vehicle heading. Let P denote the set of feasible Dubins trajectories. The overall mission trajectory is composed of N planning segments, $P=\left\{P_{1},P_{2},\ldots ,P_{N}\right\}$ , where each segment $P_{i}$ is a Dubins-feasible trajectory of the form $P_{i}={\left\{s_{k}\right\}}_{k=0}^{M_{i}}$ . The execution cost of a segment $P_{i}$ is defined according to Equation 1.

$$c\left(P_{i}\right)=\overset{M_{i}-1}{\underset{k=0}{\sum }}d_{Dubins}\left(s_{k},s_{k+1}\right),$$ (1)

where $d_{\text{Dubins}}\left(s_{k},s_{k+1}\right)$ denotes the length of the shortest Dubins curve connecting two vehicle configurations. Each segment must satisfy the budget constraint (Equation 2).

$$c\left(P_{i}\right)\leq B,\;i=1,\ldots ,N,$$ (2)

where B>0 denotes the available path-length budget for a single planning–execution cycle.

Let $I\left(P_{i}\right)$ denote a function that quantifies the information of the model along the executing path $P_{i}$ .

The informative path planning problem at cycle i is formulated as the constrained optimization problem shown in Equation 3.

$$P_{i}^{*}=\arg\underset{P_{i}\in P}{\max}I\left(P_{i}\right)\;\text{subject to}\;c\left(P_{i}\right)\leq B.$$ (3)

The overall objective is to sequentially select segments $P_{i}$ that maximize information gain under the per-cycle budget constraint, thus reducing an expected model error metric over the workspace.

## 4 Methodology

**FIGURE 1:**
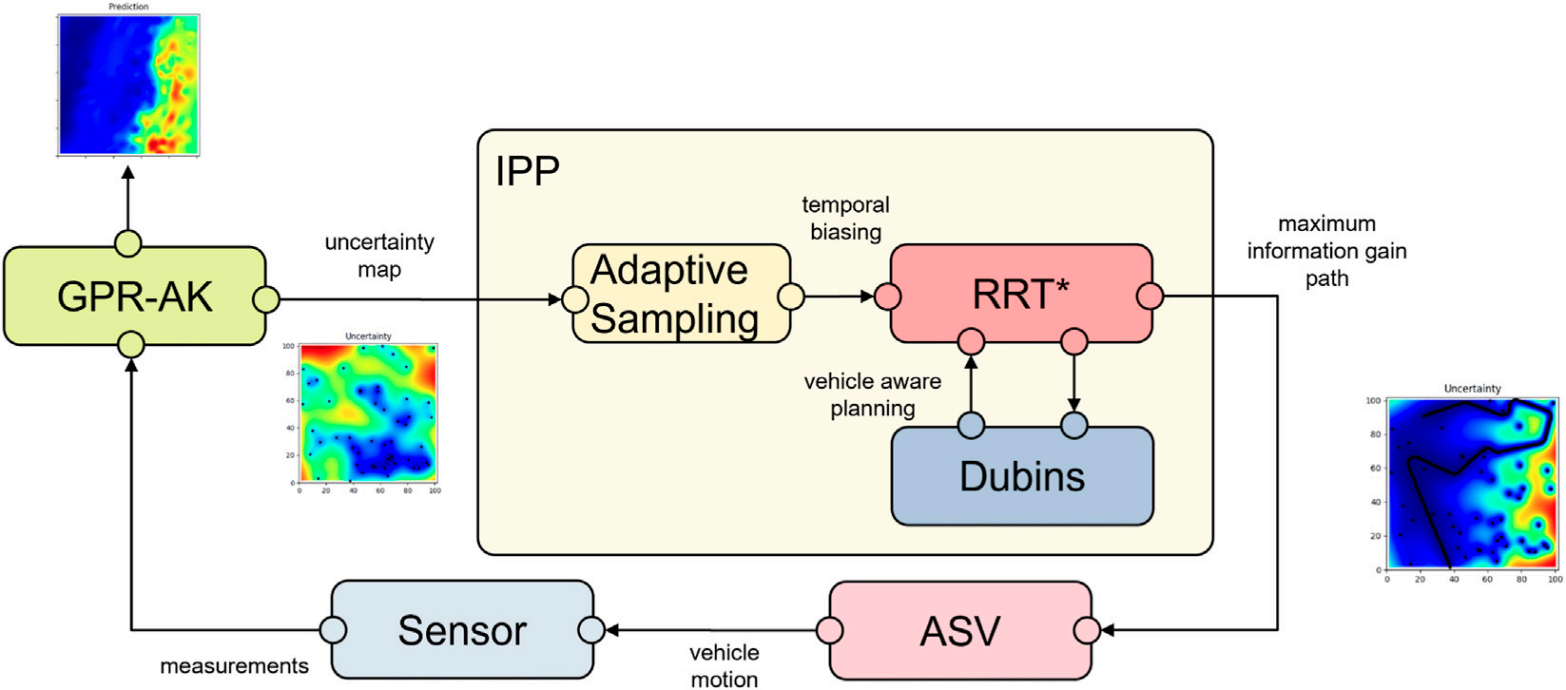
System diagram of the proposed IPP framework for an ASV. Environmental measurements collected along the vehicle trajectory are used to update a AK-GPR model, producing a spatial uncertainty map. This uncertainty representation is then used to adaptively bias the sampling process of a Dubins-constrained ${\text{RRT}}^{*}$ planner, encouraging exploration of informative regions while penalizing redundant sampling and respecting vehicle motion constraints. The resulting path is subject to a budget limit and is passed to the vehicle for execution and data collection, after which the AK-GPR model can be updated. This process can be repeated until convergence or for a predefined number of planning cycles.

The proposed approach integrates nonstationary uncertainty modeling with adaptive sampling and a modified Dubins- ${\text{RRT}}^{*}$ planner in a closed-loop framework. At each planning cycle, measurements collected along the vehicle trajectory are incorporated into an AK-GPR model, which produces predictive mean and variance estimates over the workspace. The predictive variance is interpreted as a spatial uncertainty map and is used to bias the sampling distribution of a Dubins-constrained ${\text{RRT}}^{*}$ planner. Penalization and boosting mechanisms (here, boosting denotes to a local refinement bias and is not related to ensemble boosting methods in machine learning) are introduced within this adaptive sampling layer to discourage redundant exploration and to promote refinement near high-information regions, thereby improving tree expansion efficiency. The planner generates dynamically feasible trajectories that maximize the accumulated predictive variance subject to a budget constraint. The selected trajectory is executed, new measurements are collected, and the model is updated. This process is repeated for a fixed number of planning cycles. The overall architecture of the proposed framework is illustrated in Figure 1.

### 4.1 Dubins-constrained informative ${\text{RRT}}^{*}$ formulation

The proposed planner follows a hybrid expansion–refinement strategy. At each iteration, a spatial sample is drawn from the adaptive sampling distribution $x_{\mathrm{rand}}∼p_{t}\left(x\right)$ , whose construction is described in Section 4.3.

Let F(x) denote the distance-to-obstacle function, defined as the distance from x to the nearest occupied cell or map boundary. The feasible heading $\psi_{\mathrm{rand}}$ is then selected based on F(x) . If the sampled position lies within a safety margin region $F\left(x_{\mathrm{rand}}\right)\leq \delta$ , the heading is aligned with the gradient of the distance field $\nabla F\left(x_{\mathrm{rand}}\right)$ , which steers the vehicle away from nearby obstacles or margins. Otherwise, the heading is drawn uniformly at random in [0,2π) . This yields the candidate state $s_{\mathrm{rand}}=\left(x_{\mathrm{rand}},y_{\mathrm{rand}},\psi_{\mathrm{rand}}\right)$ .

Next, a set of candidate parent connections O is generated using Dubins paths from existing tree nodes to $s_{\mathrm{rand}}$ . Candidate parents are ranked according to the path cost c(P) , defined as the cumulative Dubins length. By prioritizing shorter feasible connections, the expansion step distributes new nodes across different branches of the tree rather than greedily extending a single high-information trajectory, thereby encouraging broad exploration. A feasible connection is selected and added to the tree as a full or truncated Dubins segment depending on the remaining budget constraint c(P)≤B . This truncation mechanism introduces variability in the resulting tree structure and enables tighter utilization of the budget B .

After node insertion, a rewiring phase is performed using the accumulated information metric in Equation 4.

$$I\left(P\right)=\underset{x\in P}{\sum }\sigma_{t}\left(x\right),$$ (4)

which enables local refinement of promising trajectories while preserving the exploratory behavior induced by cost-based expansion.

Finally, the best node is selected according to a combined information and coverage criterion. Coverage is defined as the number of unique grid cells visited along the corresponding trajectory. Unlike information, coverage is not evaluated during each expansion step; instead, it is computed only when selecting the maximum-information node at the end of the planning cycle.

Unlike classical goal-directed ${\text{RRT}}^{*}$ , the proposed formulation does not rely on a goal termination condition. Instead, the planner performs a fixed number of iterations K , allowing the tree to expand broadly and efficiently within the available sampling budget. The overall procedure is summarized in Algorithm 1.

Algorithm 1. Dubins-Constrained Informative RRT*

1: Initialize tree T with root state $s_{0}$

2: Initialize bias maps: boost(x)=1 , penalty(x)=1

3: Load free-space mask F

4: **for** k=1 to K **do**
5:     Sample $x_{\mathrm{rand}}∼p_{t}\left(x\right)$

6: $\psi_{\mathrm{rand}}\leftarrow$ SELECTSAFEHEADING $\left(x_{\mathrm{rand}},F\right)$

7: $s_{\mathrm{rand}}\leftarrow \left(x_{\mathrm{rand}},y_{\mathrm{rand}},\psi_{\mathrm{rand}}\right)$

8: O← SELECTOPTIONS $\left(T,s_{\mathrm{rand}}\right)$ ⊳Rank candidate connections by cost
9:     **for** each option in O **do**
10:     **if** full extension is within budget **then**
11:     **if** extension is collision-free **then**
12:       add node with full Dubins segment
13: $s_{\mathrm{new}}\leftarrow$ inserted node state
14:       REWIRE $\left(s_{\mathrm{new}},T\right)$

15:       Update top- k nodes based on information and coverage
16:       Update penalization and boosting maps
17:       **break**
18:      **end** **if**
19:     **else** **if** truncation is feasible within budget **then**
20:      **if** truncated segment is collision-free **then**
21:       add truncated node
22: $s_{\mathrm{new}}\leftarrow$ inserted node state
23:       REWIRE $\left(s_{\mathrm{new}},T\right)$

24:       Update top- k nodes based on information and coverage
25:       Update penalization and boosting maps
26:      **break**
27:     **end** **if**
28    **end** **if**
29:  **end** **for**
30: **end** **for**

The behavior of the planner is governed by the predictive uncertainty $\sigma_{t}\left(x\right)$ , which defines both the information metric used during the rewiring and the adaptive sampling distribution. We therefore next describe the nonstationary Gaussian process model used to estimate this uncertainty over the workspace.

### 4.2 Uncertainty modeling via attentive kernels gaussian process regression (AK-GPR)

We model the unknown environmental field as a function $f_{\text{env}}:R^{D}\to R$ using GPR. In this framework, prior assumptions about the spatial structure of the field are encoded through a covariance (kernel) function that specifies the correlation between function values at pairs of spatial locations.

Standard stationary kernels, such as the radial basis functions (RBFs), assume that spatial correlations depend only on relative distance and are invariant across the workspace. Although effective in some environments, this assumption is often restrictive in real-world mapping tasks, where the spatial variability of the field can change significantly across regions. To capture such a spatially varying structure, we adopt the AK-GPR method (Chen et al., 2022b), which provides a mechanism to construct nonstationary covariance functions.

The AK expresses the covariance between two locations $x,x^{\prime }\in R^{D}$ as a location-dependent mixture of multiple base kernels as described in Equation 5:

$$ak\left(x,x^{\prime }\right)=\alpha \;z{\left(x\right)}^{⊤}z\left(x^{\prime }\right)\overset{M}{\underset{m=1}{\sum }}{\bar{w}}_{m}\left(x\right)\;k_{m}\left(x,x^{\prime }\right)\;{\bar{w}}_{m}\left(x^{\prime }\right),$$ (5)

where $k_{m}\left(\cdot ,\cdot \right)$ denotes the m -th base kernel, typically chosen as RBF kernels with different lengthscales. Normalized attention weights ${\bar{w}}_{m}\left(x\right)$ define the relative contribution of each base kernel at location x , while α is a scaling constant. The terms z(x) and $z\left(x^{\prime }\right)$ encode visibility or validity masks, allowing correlations to be downweighted in regions that are unobservable or uninformative.

This formulation enables the model to adapt locally, allowing different smoothness characteristics to dominate in different regions of the environment. As a result, the GP can represent heterogeneous spatial phenomena.

Conditioned on measurements collected at sampled locations, the AK-GPR model provides a posterior predictive mean and variance at arbitrary query locations. In this work, predictive variance is interpreted as a spatial uncertainty measure and is subsequently used to guide adaptive sampling of informative path planning. At the end of every loop, the newly collected samples are added to the training set and the AK-GPR is retrained.

### 4.3 Adaptive uncertainty-guided sampling

As the expansion behavior of sampling-based planners is strongly influenced by the sampling distribution, we next describe how predictive uncertainty is used in our planner to reshape the sampling process.

Although sampling-based planners provide scalability, uniform sampling often leads to inefficient exploration in large or partially observed environments. To address this limitation, we introduce an adaptive uncertainty-guided sampling strategy that reshapes the sampling distribution based on predictive uncertainty and planner feedback.

The adaptive bias function $b_{t}\left(x\right)$ consists of two complementary mechanisms: penalization, which discourages redundant sampling in explored regions, and boosting, which promotes refinement near high-information nodes. These components are combined to form the final sampling distribution.

Let $\sigma_{t}^{2}\left(x\right)$ denote the predictive variance of the AK-GPR model in iteration t . The sampling density is defined according to Equation 6.

$$p_{t}\left(x\right)\propto \sigma_{t}^{2}\left(x\right)\;b_{t}\left(x\right),$$ (6)

where $b_{t}\left(x\right)$ modulates the sampling distribution through these two components. Importantly, the underlying uncertainty map remains unchanged; the bias operates solely at the level of the sampling distribution.

In practice, the sampling distribution can be further adjusted by using a concentration parameter that modulates the spread of the distribution. Specifically, the sampling density may be expressed as in Equation 7.

$$p_{t}^{\left(\gamma \right)}\left(x\right)\propto {\left(\sigma_{t}^{2}\left(x\right)\;b_{t}\left(x\right)\right)}^{\gamma },$$ (7)

where γ>0 controls the concentration of the sampling distribution. Smaller values of γ produce a broader distribution that encourages exploration, while larger values concentrate sampling around the highest-uncertainty regions.

**FIGURE 2:**
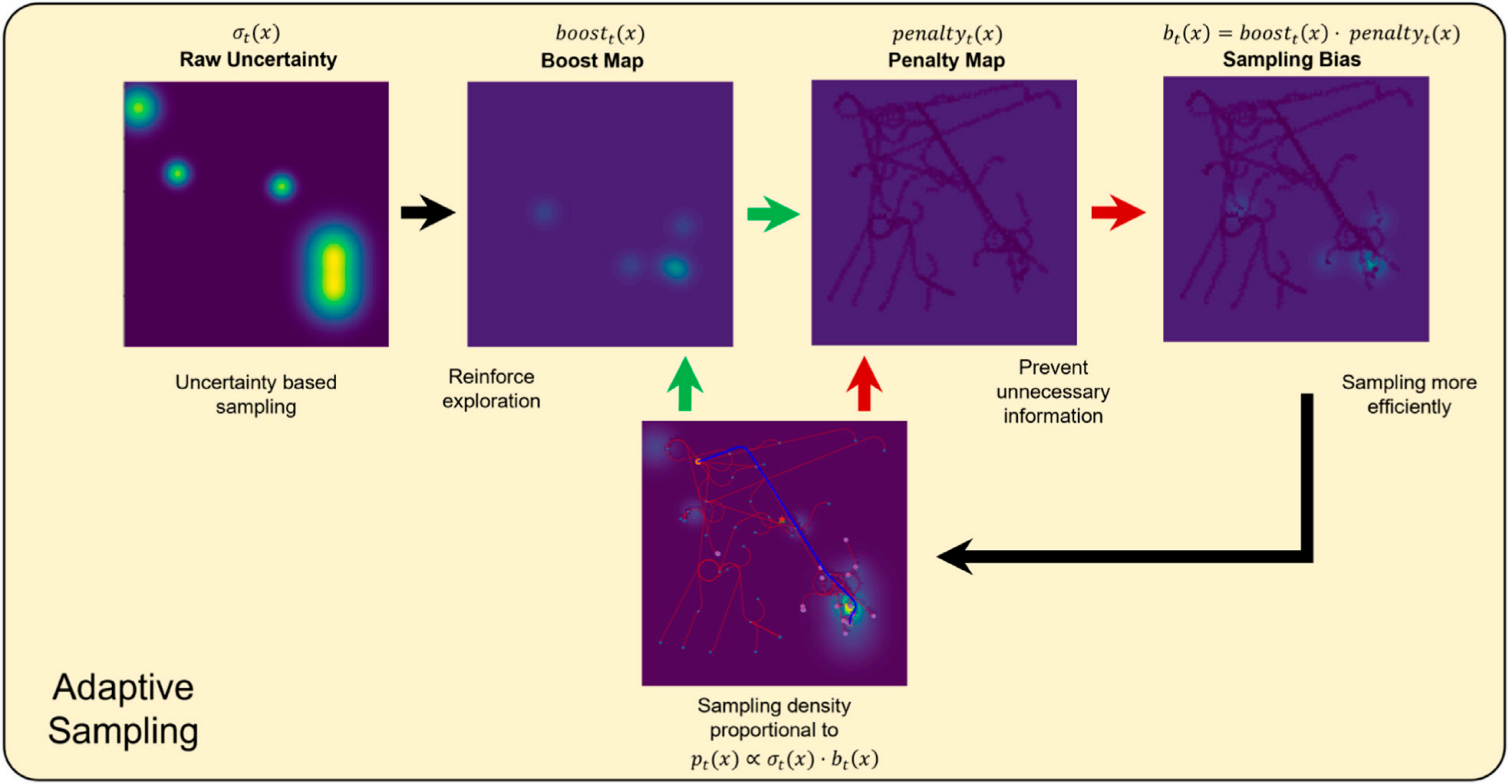
Adaptive uncertainty-guided sampling. The predictive variance $\sigma_{t}^{2}\left(x\right)$ defines the baseline sampling density. Boosting reinforces sampling near high-information nodes, while penalization suppresses sampling along previously traversed paths. The combined bias $b_{t}\left(x\right)=boost_{t}\left(x\right)\cdot penalty_{t}\left(x\right)$ reshapes the sampling distribution as $p_{t}\left(x\right)\propto \sigma_{t}^{2}\left(x\right)\cdot b_{t}\left(x\right)$ , promoting efficient and non-redundant exploration.

A schematic illustrating a sample trajectory and the construction of the bias map through penalization and boosting is shown in Figure 2.

Penalization and boosting provide complementary mechanisms to shape sampling beyond purely variance-driven strategies. The penalization map accumulates along RRT trajectories, reducing sampling probability in visited regions and discouraging redundancy. This behavior is analogous to covariance effects in mutual information-based methods, where correlated samples contribute limited additional information, but is achieved here through a lightweight, local approximation.

In contrast, the boosting map reinforces sampling near high-information nodes, promoting local refinement in informative regions. While variance emphasizes exploration, boosting introduces a controlled bias toward exploitation by focusing effort where information gain is expected to be highest. This is conceptually related to informed sampling strategies such as Informed ${\text{RRT}}^{*}$ (Gammell et al., 2014), where the search is restricted to an ellipsoidal region containing states that can improve the current solution, which shrinks as better solutions are found. In summary, penalization discourages oversampling while boosting promotes refinement, yielding a computationally efficient alternative to information-theoretic acquisition strategies by directly leveraging the GP predictive variance rather than repeatedly evaluating covariance-based uncertainty reduction along candidate trajectories. This provides a tractable surrogate for trajectory-level information gain. This is particularly advantageous in sampling-based planners, where evaluating information-theoretic objectives along candidate trajectories would otherwise become computationally costly.

#### 4.3.1 Penalization

To discourage redundant sampling in regions already explored by the planner, we introduce a multiplicative mask-based penalization map that accumulates along accepted trajectories.

Let an accepted path at iteration t be defined as $P={\left\{s_{k}\right\}}_{k=0}^{K}$ , where $s_{k}=\left(x_{k},y_{k},\psi_{k}\right)$ denotes the discretized state along the Dubins trajectory. Since penalization operates only in the spatial domain, we define the corresponding planar positions of the path as $E={\left\{x_{k}\right\}}_{k=0}^{K}$ , where $x_{k}=\left(x_{k},y_{k}\right)$ . For each position $x_{k}$ , a spatial neighborhood is defined as a ball of radius $r_{p}>0$ according to Equation 8.

$$B\left(x_{k},r_{p}\right)=\left\{\|x-x_{k}\|\leq r_{p}\right\}.$$ (8)

The set of affected spatial locations for the edge E is defined by Equation 9.

$$\tilde{E}\left(E\right)=\underset{k\in \left\{0,m,2\;m,\ldots ,\left\lfloor K/m\right\rfloor m\right\}}{⋃}B\left(x_{k},r_{p}\right).$$ (9)

Let $penalty_{t}\left(x\right)\in \left[\eta_{\min},1\right]$ denote the penalization map in iteration t , initialized as $penalty_{0}\left(x\right)=1$ . The penalization map is updated according to Equation 10.

$$penalty_{t+1}\left(x\right)=\left\{\begin{array}{cc} \max\;\left(\eta_{\min},\;\alpha \;penalty_{t}\left(x\right)\right), & x\in \tilde{E}\left(E\right),\\ penalty_{t}\left(x\right), & \text{otherwise}, \end{array}\right.$$ (10)

where α∈(0,1) controls the suppression strength in the affected region (defined by Equation 9) and $\eta_{\min}$ prevents collapse to zero (optional).

This multiplicative update causes penalization to accumulate over repeated traversals, progressively reducing the sampling probability in frequently covered regions while preserving the underlying uncertainty map.

#### 4.3.2 Boosting

To promote refinement near promising regions of the tree, we introduce a Gaussian-based boosting mechanism centered at the top- k high-information leaf nodes.

Let $L_{t}$ denote the set of top- k leaf nodes at iteration t . Each leaf corresponds to a state $s_{i}$ with spatial position $x_{i}\in R^{2}$ and accumulated path cost $c\left(P_{i}\right)$ , where $P_{i}$ denotes the path from the root $s_{0}$ to $s_{i}$ The remaining budget at that node is given by $r_{i}=B-c\left(P_{i}\right).$

For each $x_{i}\in L_{t}$ , we define a Gaussian reinforcement according to Equation 11.

$$G_{i}\left(x\right)=\exp\;\left(-\frac{\|x-x_{i}{\|}^{2}}{2\sigma_{i}^{2}}\right),\;\sigma_{i}=\frac{r_{i}}{3},$$ (11)

so that approximately 99% of the Gaussian mass lies within radius $r_{i}$ .

The combined boost field is defined by Equation 12.

$$G_{t}\left(x\right)=\underset{x_{i}\in L_{t}}{\prod }\left[1+\beta \;G_{i}\left(x\right)\right],$$ (12)

where β≥0 controls the boost strength. The boosting map is then updated according to Equation 13.

$$boost_{t}\left(x\right)\leftarrow boost_{t}\left(x\right)\;G_{t}\left(x\right).$$ (13)

Boosting is activated only after an initial number of accepted nodes, allowing the tree to expand broadly first before refinement mechanisms are introduced.

Furthermore, the boost strength is adaptively reduced when no new nodes are accepted in successive iterations. In such cases, $boost_{t}\left(x\right)$ gradually approaches 1, diminishing its influence and preventing persistent focalization in a single region of the workspace.

### 4.4 Discussion

The use of accumulated predictive variance along a trajectory serves as a practical surrogate for information gain, as regions with higher posterior variance correspond to areas where the model is less certain and thus more informative measurements can be obtained. By integrating this quantity along feasible trajectories, the planner implicitly balances exploration of uncertain regions with spatial coverage. Furthermore, the sampling concentration parameter γ modulates the exploration–exploitation tradeoff: lower values encourage broader exploration, while higher values focus sampling around high-uncertainty regions. Together, these design choices provide an intuitive mechanism for guiding informative trajectory generation under budget and kinematic constraints.

In general, the proposed framework tightly couples probabilistic uncertainty modeling, adaptive sampling, and Dubins-constrained informative path planning within a closed-loop architecture. The AK-GPR model provides a nonstationary representation of environmental uncertainty, whose predictive variance directly shapes the sampling density of the planner. Penalization discourages redundant exploration, while boosting promotes localized refinement around high-information nodes under the remaining budget constraint. The expansion of ${\text{RRT}}^{*}$ is driven by the path cost to preserve the broad exploration, and the rewiring is guided by accumulated uncertainty to improve the information gain locally. By iteratively updating the model with newly collected measurements and reshaping the sampling distribution accordingly, the framework effectively functions as a AK-GPR-driven scheduling mechanism, directing sampling effort toward regions of high residual uncertainty. In this manner, the system progressively balances exploration and refinement while respecting vehicle kinematic constraints and budget limitations.

While the proposed planner builds upon ${\text{RRT}}^{*}$ , which is known to be probabilistically complete and asymptotically optimal under uniform sampling, these guarantees do not directly carry over to the proposed framework. The adaptive sampling strategy introduces a non-uniform and time-varying sampling distribution, and the objective is defined in terms of information gain rather than path length optimality. As a result, formal guarantees on convergence or optimality are not established in this work. Instead, the method is evaluated empirically, and the planner retains desirable exploration properties inherited from sampling-based approaches while enabling more efficient information-driven behavior.

## 5 Results

Results are presented in five phases: ablation study, baseline field verification, hardware-in-the loop validation, obstacle-free mapping, and obstacle-rich mapping. First, the ablation study evaluates the contribution of the main framework components through controlled synthetic experiments. Next, AK-GPR performance is validated in the field as a baseline following a hardware-in-the-loop validation with an embedded platform (Nvidia Jetson Orin) using Gazebo. Then, results are shown for the Dubins- ${\text{RRT}}^{*}$ planner performance in an obstacle-free environment for two test scenarios: bathymetric mapping and seagrass probability mapping.

In these experiments, the proposed method is compared against several alternative planners, including standard ${\text{RRT}}^{*}$ , a myopic greedy strategy, and coverage-based baselines such as GreedyCover (Jakkala et al., 2026) and HEXCover (Dutta et al., 2022). To isolate the effect of the planning strategy, the remaining components of the framework, including the AK-GPR model, are kept consistent across methods. As many existing planners do not exactly match the problem formulation considered in this work, minor adaptations were required to enable a fair comparison.

With these adaptations, the selected baselines represent a diverse set of planning strategies, including coverage-based methods with posterior uncertainty reduction guarantees, sampling-based methods closely related to the proposed planner but without adaptive sampling, and myopic or greedy local decision strategies. Finally, results for the planner are presented in both obstacle-free and obstacle-rich environments. The obstacle-free experiments include bathymetry and seagrass mapping scenarios, while the obstacle-rich experiments include synthetic mapping environments and algal plume mapping scenarios.

The purpose of the field deployment is to validate the feasibility and real-world consistency of the AK—GPR modeling component and sensing pipeline. Due to operational constraints, full closed-loop evaluation of the Dubins–RRT* planner is conducted in simulation, where controlled comparisons across multiple planners and environments can be performed.

### 5.1 Ablation study

**FIGURE 3:**
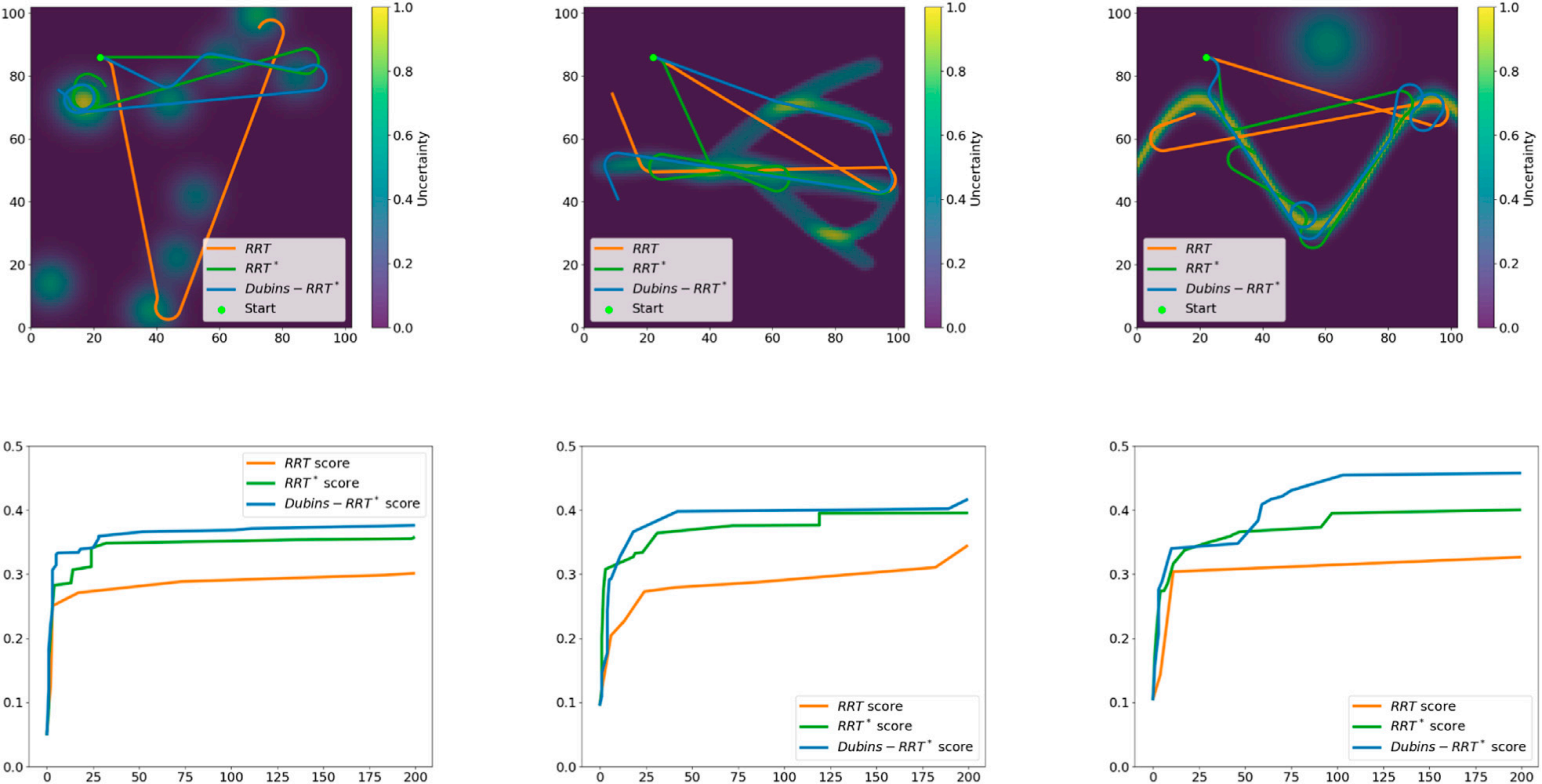
Ablation study comparing RRT , $RRT^{*}$ , and Dubins– $RRT^{*}$ (ours) across three synthetic environments. Columns from left to right correspond to Scenario 1 (sparse localized uncertainty regions), Scenario 2 (branched informative corridor), and Scenario 3 (curved uncertainty ridge). In each column, the top image shows the final path obtained by each planner over the uncertainty field, while the bottom image shows the corresponding score evolution over planning iterations. All planners use the same Dubins-constrained motion model, budget (200 m) and number of iterations (200). Across all scenarios, standard RRT obtains the lowest score due to the absence of rewiring or adaptive sampling. Incorporating rewiring through $RRT^{*}$ improves path quality and final information gain. Dubins– $RRT^{*}$ consistently achieves the highest score across all scenarios by combining rewiring with adaptive uncertainty-guided sampling.

To better illustrate the contribution of each component of the informative path planner, we isolated the sampling and planning modules from the full framework and evaluated their effects independently. All planners evaluated in this ablation study employ the same Dubins-constrained motion model and budget constraints. The primary differences between variants arise from the use of rewiring and adaptive uncertainty-guided sampling. The goal of this section is to compare RRT , $RRT^{*}$ , and the proposed Dubins– $RRT^{*}$ planner in order to understand the improvements introduced by rewiring and adaptive sampling. In this setup, all planners use the same budget (200 m) and are allotted the same number of iterations (200). In order to emphasize different exploration challenges, three separate synthetic environments were designed with specific uncertainty distributions. Scenario 1 represents a sparse localized uncertainty environment, while Scenario 2 presents a branched informative structure, and Scenario 3 encapsulates a smooth curved ridge. The resulting paths and score evolution are shown in Figure 3.

The results in Figure 3 show a consistent ranking across all three scenarios. Standard RRT produces the lowest scores, which is expected since it lacks a rewiring mechanism and therefore tends to preserve early suboptimal branches. Although it can eventually reach informative regions, its final solution is strongly influenced by the order in which samples are added, leading to paths that are less aligned with the structure of the uncertainty field. Introducing rewiring with $RRT^{*}$ improves performance in all cases, as reflected by the higher score curves and the smoother final paths. This confirms that, even without adaptive sampling, local refinement of the tree helps concentrate the trajectory around more informative portions of the environment.

The Dubins– $RRT^{*}$ variant achieves the best performance in the three synthetic scenarios. Its advantage is especially clear in Scenarios 2 and 3, where the informative regions follow structured continuous patterns rather than isolated peaks. In these cases, adaptive sampling guides the growth of the tree toward the most promising parts of the map. As a result, the planner not only reaches informative areas earlier, but also remains better aligned with the geometry of the underlying uncertainty distribution.

The score trajectories further indicate that most of the gain is obtained during the first iterations, after which all methods tend to settle; however, the settling point reached by Dubins– $RRT^{*}$ is consistently higher. This behavior suggests that rewiring contributes to solution refinement, while adaptive informative sampling is responsible for accelerating convergence toward high-value trajectories. As with many sampling-based planners, increasing the number of iterations generally reduces the performance gap between methods, since additional samples improve tree coverage and refinement. However, convergence properties depend on the specific planner, rewiring strategy, and sampling distribution.

**FIGURE 4:**
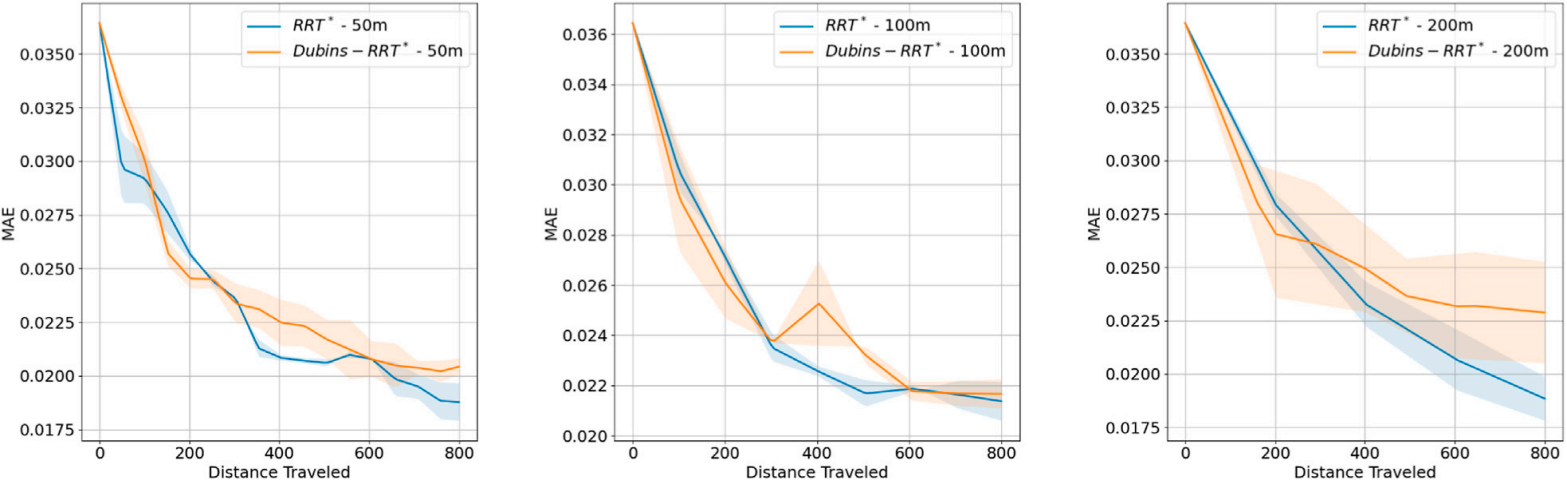
Cross-experiment comparison between $RRT^{*}$ and Dubins– $RRT^{*}$ under different planning budget settings. From left to right, the panels correspond to budgets of 50 m , 100 m , and 200 m , respectively. In all cases, the maximum travel distance, samples, and number of planning iterations were kept fixed, such that the main effect of increasing the budget is a reduction in replanning frequency and AK-GPR retraining events. The results show that planner performance depends on the tradeoff between frequent model updates at smaller budgets and longer planning horizons at larger budgets.

To further evaluate the impact of the AK-GPR module within the framework, we compared $RRT^{*}$ and Dubins– $RRT^{*}$ under different planning budget settings of 50 m , 100 m , and 200 m while keeping the maximum travel distance fixed. Under this setup, the total sensing effort remains the same, while the number of replanning cycles and AK-GPR retraining events varies with the selected budget. By analyzing the performance gap in terms of the MAE between both planners across these settings, shown in Figure 4, we can better understand the contribution of the GPR module within the overall approach.

The budget ablation highlights a tradeoff between planning horizon and replanning frequency, resulting in a negative correlation between planning budget and final reconstruction error. Since both methods are Dubins-constrained and the total travel distance, sensing effort, and planning iterations are kept fixed, the primary factor affected by the budget is the frequency of replanning and AK-GPR updates. As observed in Figure 4, smaller budgets divide the mission into shorter segments, leading to more frequent posterior updates and allowing both planners to react more often to newly acquired measurements. In this case, AK-GPR more strongly dominates the resulting trajectory, and the outcome becomes less influenced by the planner itself. The performance gap between Dubins– $RRT^{*}$ with adaptive sampling and its non-adaptive counterpart becomes smaller, and can even reverse in some cases. As the budget increases, replanning becomes less frequent, and thus the planners have a larger influence over the resulting trajectories. These results suggest that the contribution of adaptive sampling is strongly coupled to replanning frequency, with its advantage becoming more pronounced when the planner must commit to longer trajectory segments between GP updates. A key takeaway is that planner performance is fundamentally coupled with model update frequency, revealing an inherent tradeoff between frequent uncertainty adaptation under short budgets and increased planner influence under longer planning horizons.

Overall, the ablation studies confirm that the proposed framework benefits from the complementary interaction between planning and learning components. Rewiring improves path quality over standard RRT , while adaptive sampling further increases information gain by directing exploration toward high-value regions. In addition, the budget analysis shows that AK-GPR update frequency plays a key role in reconstruction performance, revealing a tradeoff between frequent model adaptation and longer planning horizons. These results indicate that the planning budget should be carefully selected to balance reconstruction accuracy, planner influence over the resulting trajectories, and the computational cost associated with repeated GP retraining.

### 5.2 Datasets and environments

To facilitate the use of real-world data, a virtual sensor is employed that generates a measurement based on image data. To test the performance of the proposed Dubins– ${\text{RRT}}^{*}$ planner with the AK-GPR, three different environments are proposed. These three applications span a variety of environmental mapping applications and were selected for complexity, variety, and impact. The three applications are bathymetry, seagrass surveying, and algal plume monitoring.

**FIGURE 5:**
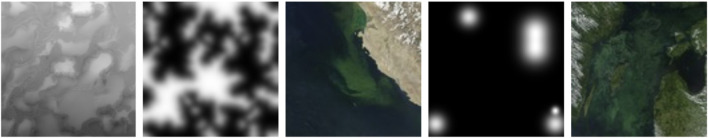
Mosaic of test environments. (L–R) Gulf of Mexico bathymetric imagery (National Centers for Environmental Information NCEI, 2026), seagrass probability mask, Baltic Sea satellite algae imagery (bal, 2005), synthetic test environment, Peru coastal satellite algae imagery (per, 2004).

To construct these environments, images were mapped to the defined test area and masked to account for the shore. Temporal variation is not considered under the assumption that variation over the course of a trial is negligible. These environments represent distinct applications of the proposed algorithm. The differences between test environments include different sensor suites, different bodies of water, and different vehicles. As summarized in Table 1, these three applications model three types of measurement and may or may not include obstacles. There is a critical difference in the model output. Algal plume mapping and bathymetric surveying generate a continuous depth or concentration map, while seagrass mapping generates a probability map. The successful implementation of a versatile solution must work in both environments. The purpose of introducing both test environments is to demonstrate that the algorithm is sensor-agnostic and vehicle-independent. All environments are modeled as two-dimensional mapping problems. The test environments are summarized in Figure 5.

**TABLE 1:** Environmental datasets and measurement characteristics.

Environment	Measurement type	Obstacle presence
Bathymetric profile	Depth	No
Seagrass	Probability	No
Algal plumes	Concentration	Yes

The first environment and configuration correspond to bathymetric surveying. The test data were extracted using the NCEI Grid Extract tool (National Centers for Environmental Information NCEI, 2026), specifically from the Multibeam Bathymetry Mosaic layer, which provides 32-bit floating-point values representing the depth of the seafloor. The dataset has a spatial resolution of 3 arcseconds (approximately 90 m). This setting is modeled as a two-dimensional mapping problem using a point sensor to emulate a sonar measurement at each sampled location. Because the dataset corresponds to an open-water region without navigational constraints, no obstacles are imposed on the environment.

**FIGURE 6:**
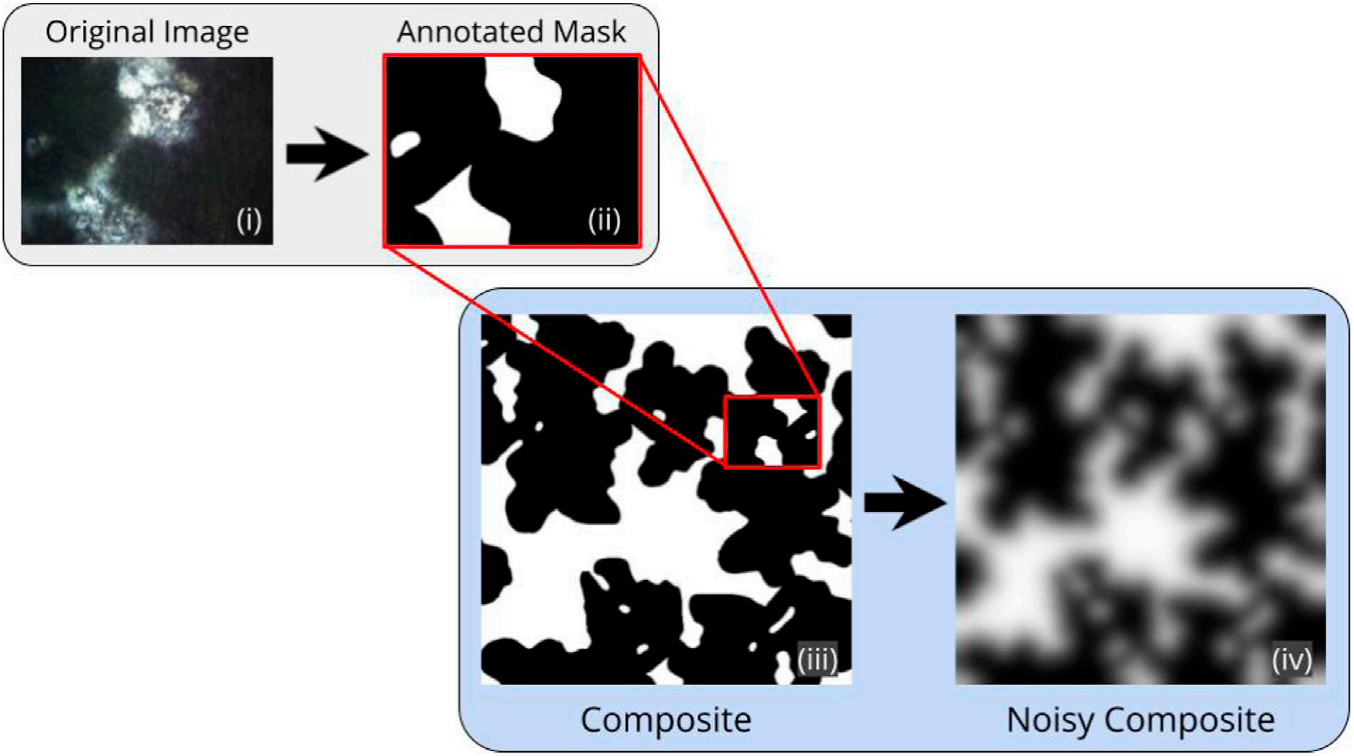
Seagrass data. (i) Sample imagery of seagrass (Martin-Abadal et al., 2018); (ii) annotated mask, where black designates occupancy and white indicates seagrass-free area; (iii) composite image of many sample seagrass images, with the position of a sample tile (rotated) shown in red (Martin-Abadal et al., 2018; Rajani et al., 2025); (iv) blurred image to model probability map.

The second dataset consists of seagrass images. Pre-segmented images were used to test overall efficacy of the mapping algorithm, as the image segmentation algorithm itself is not within the scope of this work. Base images were obtained from Rajani et al. (2025) and Martin-Abadal et al. (2018) and annotated by hand. Here, the camera measurement at a certain point was simulated as a point measurement, where the point is the center of the collected image data at a given position. A composite testbed, as shown in Figure 6, was created from multiple images to capture a variety of shapes and expand the spatial extent of the environment. This composite map is post-processed to include blurring as a representation of a probabilistic map, rather than purely binary data. This is done in accordance with the typical output of such seagrass image processing pipelines.

Finally, in the obstacle-rich portion of this work, modeling focuses on plume mapping, specifically algal plume mapping. A synthetic environment is introduced for the baseline testing. The synthetic map is generated by superimposing multiple Gaussian components at different locations with different amplitudes and spreads, producing localized high-intensity regions distributed across the workspace. The peaks are positioned near different corners to enforce spatially distributed exploration rather than concentrated sampling in a single area. An obstacle mask is then overlaid to introduce non-trivial navigation and verify the planner’s ability to balance information gain with collision avoidance and kinematic feasibility. Then, this was expanded to algal plumes. Algal plumes were selected for their environmental impact, ubiquity, and complexity. The algal plume simulation data comes from two sources. Although not specifically algae, in preliminary and baseline field testing aerial plume images from Fatchurohman et al. (2021), which consist of tracer dye in rip currents. These are included for their highly irregular, patchy shapes, which mimic the complex shape of algal plumes. Additional test data of aerial algal blooms come from the NASA Earth Observatory MODIS dataset (Bright Waters off Peru, 2004; Summer Bloom in the Baltic Sea, 2005) and noise from obstruction (e.g., clouds) is removed as appropriate. Here, the sensor is modeled as a point sensor to emulate a fluorometer. Obstacles are applied in the form of a mask over the land portions, marking non-water areas.

### 5.3 Stage I: AK-GPR deployment and feasibility

#### 5.3.1 Field deployment and simulation consistency

To establish a baseline for the AK-GPR performance without the Dubins-constrained ${\text{RRT}}^{*}$ planner and establish its feasibility, the initial AK-GPR algorithm was implemented in Gazebo and onboard an ASV. Instead of using the AK-GPR ${\text{RRT}}^{*}$ planner to generate waypoints, they were selected using a myopic approach from a set of candidates based on the maximum normalized value of the predictive entropy and the distance to the current vehicle location. 20 points were randomly sampled from the field to initialize the model, and intermediate samples are collected between waypoints at a fixed interval.

The purpose of this phase of testing was to confirm that the performance of the vehicle and the algorithm in the field is consistent with the performance of the vehicle in simulation. The test site was selected based on both the size of the water body and accessibility. The chosen location, Lake Fairfield in Lafayette, Indiana, is sufficient for vehicle maneuvering and includes boat ramps necessary for safe deployment and recovery. The vehicle selected for this deployment is the WAMV-16 (Ocean Power Technologies) with control implemented using the Robot Operating System (ROS). This vehicle has three onboard computers. Two of these are NVIDIA Jetson AGX Orin and one is a Raspberry Pi. The Raspberry Pi, a simpler microprocessor, handles low-level control, while the more computationally advanced Jetson computers handle higher-level control. A multi-processor setup is employed since the hardware is prepared for multipurpose operations; however, the full computational power is not required, and all relevant planning and model updates are executed on a single Jetson Orin, demonstrating the feasibility of real-time operation on embedded hardware.

**FIGURE 7:**
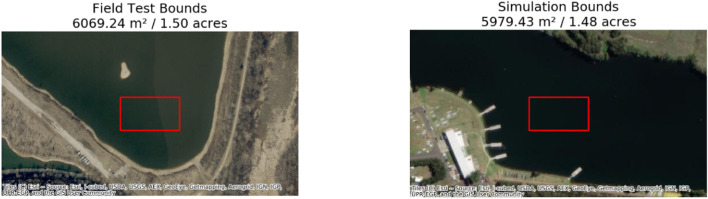
Testing area at Lake Harner overlaid onto satellite imagery compared to the simulated testing area.

For algorithm verification, a simulated sensor was used to introduce a ground truth for direct comparison of the work in simulation with the performance of the vehicle. A test image from the prior set was overlaid in the test area as shown in Figure 7, with the GPS coordinate bounds displayed in Table 2. This area was selected based on the known bathymetry of the lake for vehicle safety. A test area of equivalent size was employed in simulation trials in Gazebo using the VRX simulation suite developed by Bingham et al. (2019) to compare results between field and simulation trials. Trials were started from the same point for every run and the same number of waypoints, 20, was used between simulation and field work.

**TABLE 2:** Coordinate testing bounds.

Latitude	Longitude
40.44815	−86.86880
40.44867	−86.86756

**FIGURE 8:**
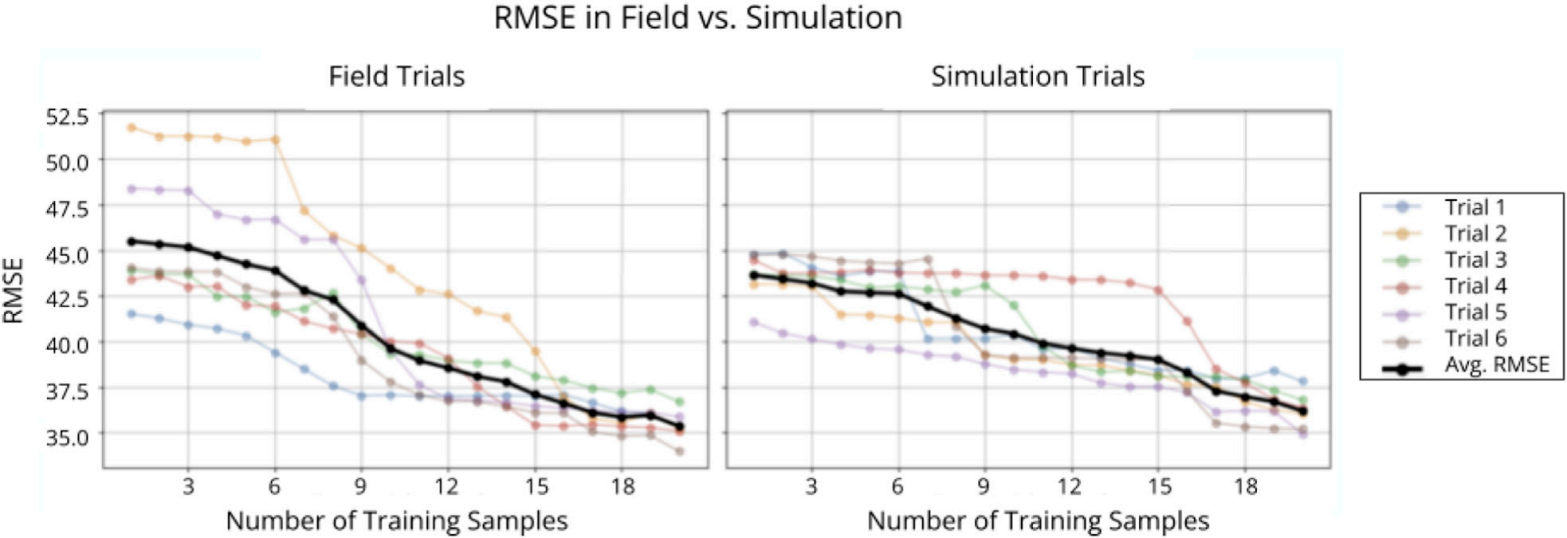
Error evaluation for 20-point mission for (L) field trials and (R) simulation trials. Both mission types converge to similar values.

Congruence between the field results and simulation results, as demonstrated by convergence of the total spatial RMSE, demonstrates that the algorithm performance translates effectively to real-world deployment. This is presented in Figure 8. The larger initial spread observed in the field results may be attributed to variations in starting locations, environmental disturbances, and slight deviations from the nominal trajectory executed in simulation.

**FIGURE 9:**
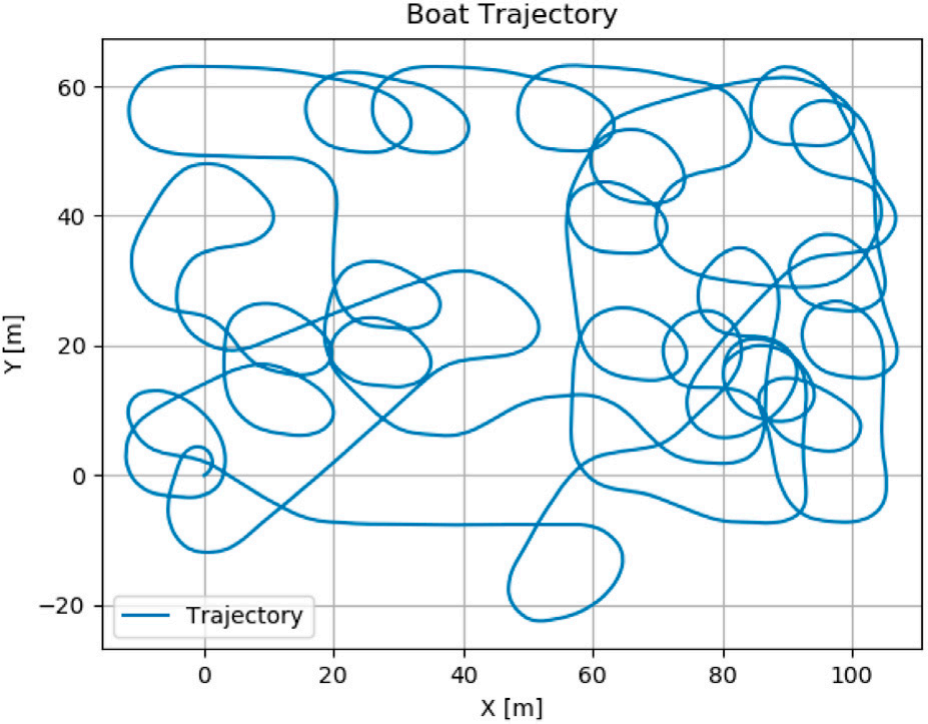
WAMV trajectory in Lake Harner, showing point-by-point planner with Dubins constraints.

The trajectory of the vehicle during a representative field trial, shown in Figure 9, motivates the changes implemented in the informative path planner relative to the baseline planner used in field testing. The baseline planner queues a single waypoint at a time using a myopic greedy strategy that targets locations based solely on local information criteria. Due to its point-by-point nature, the resulting path is not efficient, as evidenced by extraneous loops in the trajectory. Despite this inefficiency, the trajectory illustrates the need to integrate Dubins-constrained motion directly into the planning formulation, rather than targeting single locations.

While the field experiments demonstrate qualitative agreement with simulation results, several real-world factors influence system performance and highlight the importance of incorporating physical constraints directly into the planning framework. In particular, trajectory deviations arise from environmental disturbances such as wind and surface currents, as well as localization noise from GPS measurements, which introduce discrepancies between planned and executed paths. These effects contribute to the variability observed in the field trials compared to simulation, as reflected in the broader spread in error convergence. Additionally, the point by point planning strategy used in the baseline implementation amplifies these inefficiencies, as local decisions do not account for accumulated trajectory cost or disturbance-induced deviations. This reinforces the need for trajectory-level planning approaches, such as the proposed Dubins-constrained framework, which generate smoother and more robust paths under real-world operating conditions.

Based on this testing, a more effective planner would generate an entire trajectory rather than a single waypoint by jointly optimizing the sampling location and accumulated information under budget constraints, thus producing smoother and more informative trajectories. The informative Dubins– ${\text{RRT}}^{*}$ presented in this work addresses this need. In contrast, the budget-based myopic planner used as the baseline planner in later results also incorporates Dubins feasibility and budget constraints, but selects target locations based solely on local information rather than accumulated trajectory gain. Furthermore, the myopic algorithm is not sampling-based; instead, it selects the goal location from an evenly distributed grid of candidate sampling locations.

#### 5.3.2 Hardware-in-the-loop bathymetric experiment

After identifying the need to move from a point-based following strategy to a more holistic approach that accounts for vehicle motion, the proposed method was first implemented in simulation. The implementation was developed in Python within a ROS2 framework, which allows ease of integration with existing robotics tools. Initial experiments demonstrated good performance and real-time feasibility. One important observation is that planning constitutes the main computational bottleneck of the approach. However, by adopting a multithreaded implementation, it is possible to structure the system as a staged pipeline, allowing continuous execution and practical real-time operation.

Initial simulation experiments were conducted on a computer equipped with an Intel Core Ultra 9 285HX (24 cores) and an NVIDIA RTX PRO 4000 Blackwell Laptop GPU. The GPU was not utilized, as it does not provide significant advantages for the current implementation. Additionally, although the platform provides a large number of CPU cores, not all components of the pipeline are parallelizable, limiting full hardware utilization.

**FIGURE 10:**
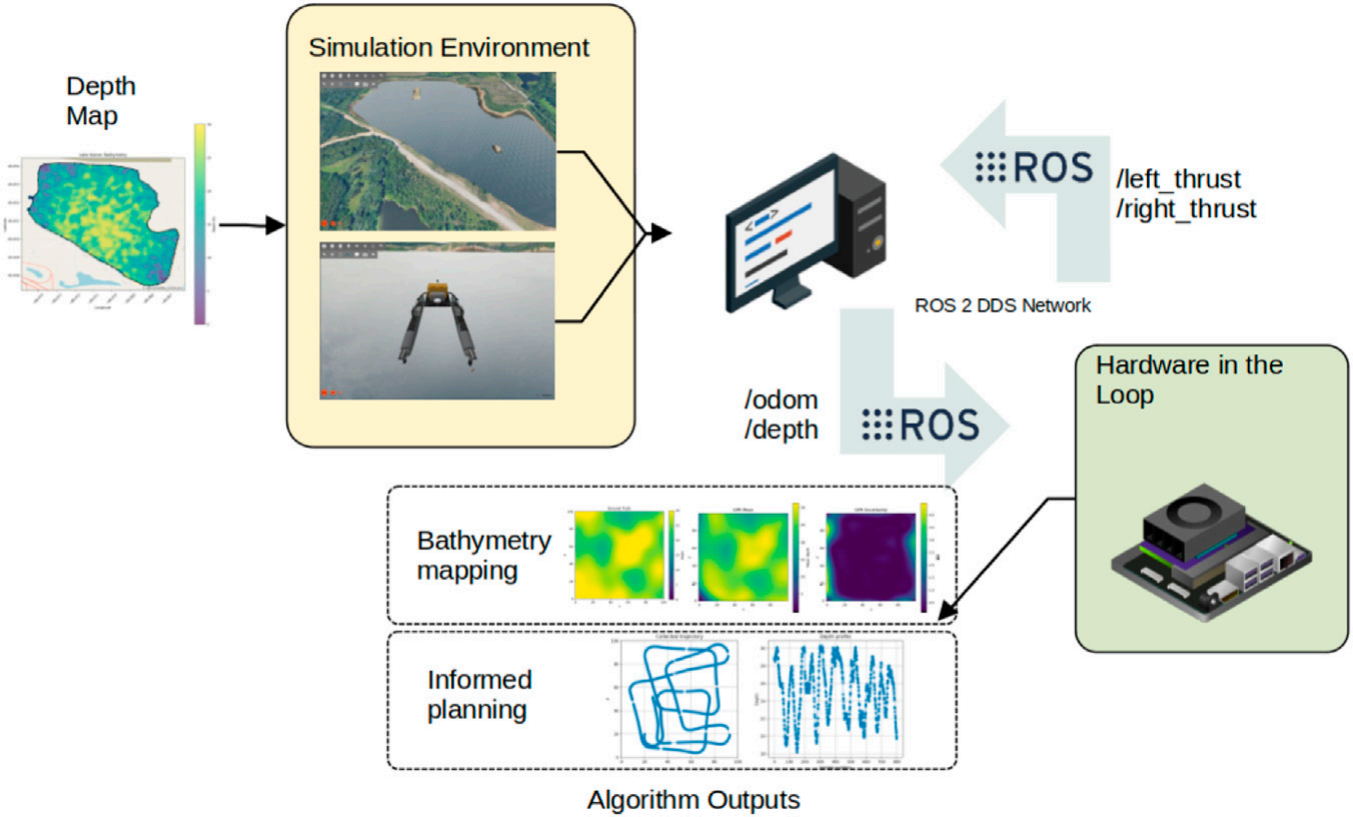
Hardware-in-the-loop experimental setup integrating a simulation environment with onboard computation through an embedded platform.

To move toward a more realistic deployment scenario, a multi-computer setup was implemented, as shown in Figure 10. In this configuration, an NVIDIA Jetson Orin Nano was used to execute the onboard components of the system, enabling hardware-in-the-loop validation.

**FIGURE 11:**
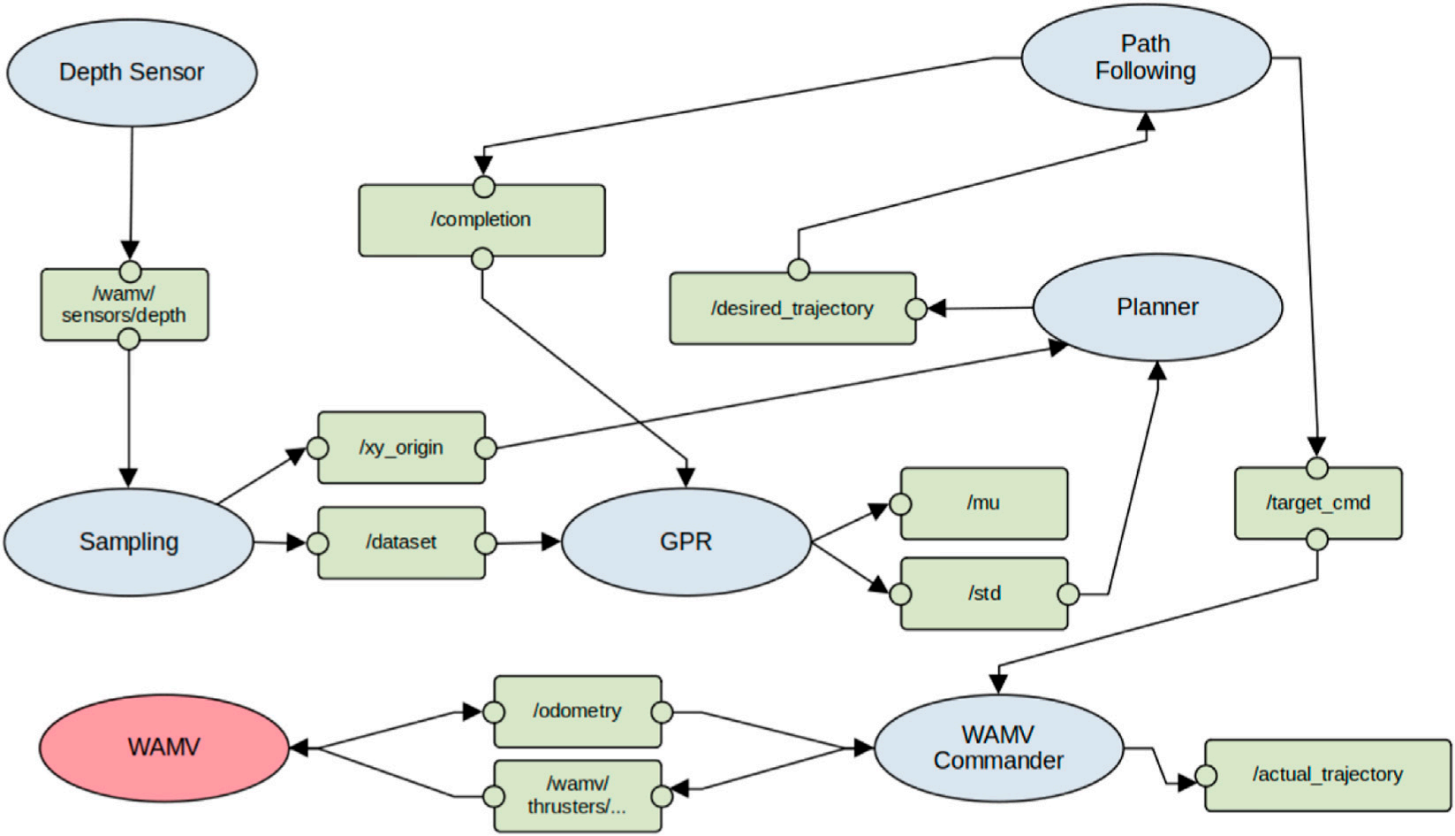
Conceptual ROS2 based software architecture illustrating the modular organization of planning, data acquisition, model update, path following, and control components. Low-level implementation details are intentionally omitted to emphasize system-level interactions and improve clarity of presentation.

The simulation environment was based on the Gazebo VRX simulation, extended with custom scenarios incorporating a WAM-V platform and environmental disturbances such as wind and waves. The overall system was implemented as a set of ROS2 nodes, as illustrated in Figure 11.

As previously noted, the planning stage dominates the computational cost. In the current implementation, nearest-neighbor and rewiring operations are performed through linear scans over the node set, resulting in a complexity that scales linearly with the number of nodes. Although this design was selected for simplicity, these operations can be improved to logarithmic complexity by incorporating spatial indexing structures such as k-d trees, without modifying the overall framework.

It is important to note that the computational cost of Gaussian Process (GP) regression scales cubically with the number of training samples due to matrix inversion operations. While the Attentive Kernel (AK) improves the efficiency of kernel computation, with complexity scaling as $O\left(N^{2}D\right)$ (Chen et al., 2022b), the overall cost of GP training remains dominated by the cubic term. This can limit real-time applicability in large scale missions.

In the proposed framework, this limitation is mitigated by controlling the effective dataset size through spatial partitioning and bounded exploration. In particular, the use of local regions (tiles) and a finite planning budget limits the number of samples contributing to each GP model, maintaining tractable update and inference times.

This introduces a trade-off between mapping resolution, model fidelity, and computational cost. Higher spatial resolution and larger coverage areas increase the number of samples required, leading to higher computational demands. Conversely, localized models and bounded sampling reduce computational cost at the expense of spatial detail. In practice, these parameters can be adjusted according to the computational capabilities of the target platform, enabling real-time deployment on embedded systems such as marine robots.

To address scalability to larger environments, the approach can be extended by partitioning the operational area into smaller tiles, allowing independent GP models to be maintained locally. This strategy bounds the size of each GP, reducing computational complexity while enabling the system to scale to large mapping areas without compromising performance, consistent with prior work addressing GP scalability limitations (Masaba et al., 2024).

We additionally evaluated the computational performance of the framework during the hardware-in-the-loop experiments. For an operational area of approximately 100 m×100m , each planner iteration requires approximately 170 m on the embedded platform. Planning is performed with a bounded budget of 50 m, and data is collected at a rate of 1 Hz, resulting in approximately 50 new samples per planning cycle.

The computational cost of GP updates increases as the dataset grows, with observed training speeds decreasing from approximately 90, 60, 50, to 47 iterations/s over successive cycles. While this reflects the expected increase in computational cost with larger datasets, the effective dataset size remains bounded through overall mission budget, spatial partitioning and mission constraints. In practice, planning and model updates remain within the available time budget, enabling continuous real-time operation throughout the mission.

**FIGURE 12:**
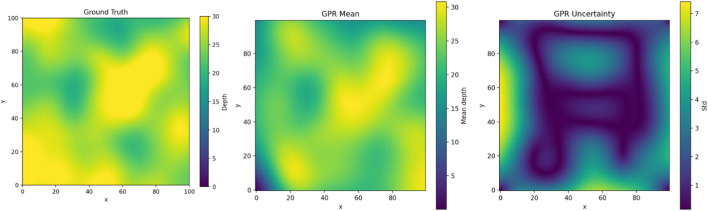
Comparison of the ground truth bathymetric map (left), the reconstructed map obtained from the proposed method (center), and the corresponding uncertainty estimate (right). The reconstruction captures the main spatial structures of the environment, while the uncertainty highlights regions with limited observations. The results correspond to a mission with a total travel budget of 350 m. The onboard computation of the algorithm was performed on an embedded platform (NVIDIA Jetson Orin Nano), while the simulation environment was executed on a workstation equipped with an Intel Core Ultra 9 processor.

Figure 12 provides a qualitative comparison between the ground truth bathymetric map, the reconstructed map, and the associated uncertainty. Despite the limited number of transects, the proposed method captures the dominant spatial structures of the environment, including large-scale gradients and localized variations. This indicates that the approach effectively prioritizes informative regions, enabling accurate reconstruction with reduced sampling effort. The corresponding uncertainty map reflects the spatial distribution of measurements, with higher uncertainty concentrated in underexplored areas, further illustrating how the model supports targeted exploration under sparse, sequential sensing conditions.

### 5.4 Dubins– ${\text{RRT}}^{*}$ - AK-GPR testing conventions

For all tests in Stage II and Stage III, conditions are held constant across trials in a given environment and experiment unless specifically noted. A set of five starting points is maintained standard across each experiment, along with three different initial grid sampling points. The total budget for all tests is 800 m , with an iteration budget of 100 m . Planner parameters remain constant across trials, and the same random seed is used across all experiments to facilitate reproducibility. All planners were implemented in Python, and the AK-GPR model was used from its original library without modification. Throughout the results section, the term IPP is used interchangeably to refer to the proposed Dubins– ${\text{RRT}}^{*}$ planner.

A grid of points is used to initialize the system. This mimics the use of coarse satellite imagery to initialize the operation of a surface vehicle or a rough sonar survey for undersea measurements such as seagrass. This convention is used throughout the testing of the Dubins– ${\text{RRT}}^{*}$ - AK-GPR planner. Different grid sizes, corresponding to the sampling density, are used to test different sizes of initialization datasets. The grid size defines the number of points sampled in the x - and y - direction within the test environment. For example, a 5×5 grid results in a 25-point initialization of the model. In test cases that include obstacles, invalid initialization points are removed and not resampled. For example, if 3 of the generated grid points fall within a blocked area, a 5×5 grid will result in 22 initialization points. To emphasize that the base sampling method is a regular grid, this is still labeled as a 5×5 grid. “Grid size”, as used in this paper, refers to this density (e.g., 5×5 ).

The Dubins turning radius is chosen as the minimum allowable value compatible with the 100×100 testbed resolution. The same testbed size was used across environments for parameter consistency. The noise of the virtual sensor is selected to be 3% of the total measurement range for that environment; in the case of obstacle-enabled environments, the sensor noise is tuned in accordance with the environment range after masking. All environments are normalized to produce ground truth measurements pre-masking within the range [0,1].

For all cases, the performance evaluation of the algorithm is conducted using two metrics. The first is the mean absolute error (MAE) to quantify model accuracy. To evaluate the uncertainty of the GP model, the trace of the covariance matrix P is used.

All quantitative results are based on five independent trials and are reported as mean ± one standard deviation. These values provide a descriptive measure of variability and are not intended as formal statistical significance tests. Accordingly, the reported trends should be interpreted as indicative rather than statistically conclusive.

### 5.5 Stage II: Dubins– ${\text{RRT}}^{*}$ - AK-GPR evaluation (obstacle-free)

After establishing the feasibility of the AK-GPR as a viable option for mapping in the field, obstacle-free mapping is presented to evaluate algorithm performance. The real-world analogue of this testing is open-water monitoring efforts. Here, bathymetric modeling and seagrass mapping are selected as representative applications, as both can be realistically abstracted to an open-water environment.

The purpose of this section is to evaluate the performance of the Dubins– ${\text{RRT}}^{*}$ - AK-GPR planner in an obstacle-free environment. A comparison against several baseline methods, including a myopicplanner, RRT ∗ , and coverage based approachessuch as GreedyCover, and HEXCover in order to justify the selection of the Dubins– ${\text{RRT}}^{*}$ - AK-GPR planner using bathymetric data. Then, after comparing the proposed method against these alternative planners, the framework is expanded to seagrass mapping to demonstrate versatility.

#### 5.5.1 Bathymetry and grid density evaluation

Bathymetric mapping data from a large open-ocean setting are selected as a first test to act as an initial testing point to evaluate performance and compare the outcome between the Dubins– ${\text{RRT}}^{*}$ - AK-GPR planner and the baseline methods under different initialization conditions.

**FIGURE 13:**
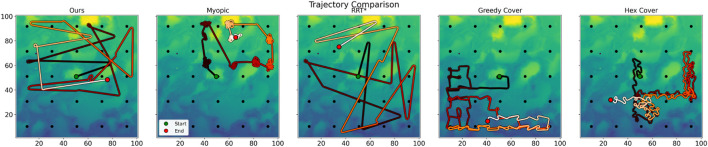
Comparison of trajectories for (left to right) Dubins– ${\text{RRT}}^{*}$ , myopic, ${\text{RRT}}^{*}$ , GreedyCover, and HEXCover approaches in the obstacle-free bathymetry environment for a total run distance of 800 m . The initialization point grid is shown in black. The start of each trajectory is depicted by a green dot, the progression of the trajectory is illustrated by a color gradient, and the end of the trajectory is depicted by a red dot. The trajectories are plotted over the ground truth.

Presented first is a comparison of an example trajectory for baseline planners and an example trajectory for the informative Dubins– ${\text{RRT}}^{*}$ - AK-GPR planner to demonstrate the difference in exploration strategies. This is represented in Figure 13. These trials reach the same final distance (800 m) , begin at the same set of starting points, utilize the same free-space sampler, begin with the same GP parameters, and have the same episodic budget (100 m) .

The Dubins– ${\text{RRT}}^{*}$ planner achieves higher coverage and distributes sensing effort more effectively across the environment than the myopic planner, standard ${\text{RRT}}^{*}$ , GreedyCover, and HEXCover approaches. While the myopic method prioritizes immediate local information gain, resulting in concentrated sampling around nearby high-value regions, the proposed planner seeks informative trajectories over a finite horizon and balances information gain with exploration. Standard ${\text{RRT}}^{*}$ exhibits good coverage; however, it lacks informative guidance, leading to less efficient use of the travel budget. GreedyCover and HEXCover emphasize uncertainty reduction guarantees, but their trajectories become constrained by local candidate selection and fragmented coverage patterns. In contrast, the informative Dubins– ${\text{RRT}}^{*}$ planner maintains smoother long-range exploration while still revisiting relevant areas, which is particularly advantageous under sparse initialization when large portions of the environment remain unknown.

**FIGURE 14:**
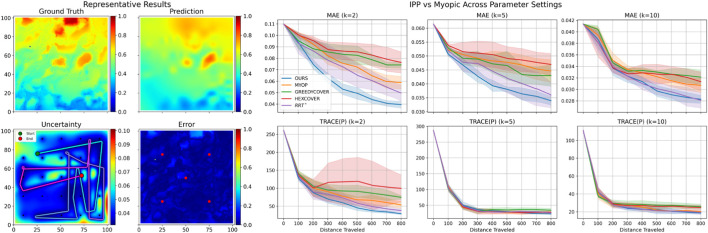
(Left) Representative results for bathymetric mapping for a total distance of 800 m and starting grid of 5×5 , showing (clockwise from top left): the ground truth, the prediction, error (absolute) with starting points for all trials, and uncertainty (σ) overlaid with robot path for a sample Informative Dubins– ${\text{RRT}}^{*}$ ) trial. (Right) MAE and Tr(P) results comparing the baseline planners and our proposed approach (Informative Dubins– ${\text{RRT}}^{*}$ ), corresponding to different initialization parameters in the bathymetric mapping environment. Results reflect a starting grid of 2×2 , 5×5 , and 10×10 . These metrics are overlaid with ±1σ based on n=5 trials.

This difference in strategies is further visible in the results presented in Figure 14, with final tabulated values in Table 3. For all planners and across the three initialization grid sizes, there is a downward trend in the MAE, demonstrating improved model accuracy as more data is added. As expected, model accuracy improves with a denser initialization grid, as the model fit is based on more data. The informative Dubins– ${\text{RRT}}^{*}$ planner generally achieves lower MAE across trials and tends to converge faster than the baseline methods, including the myopic planner, standard ${\text{RRT}}^{*}$ , GreedyCover, and HEXCover. This suggests that the proposed planner may use the travel budget more effectively. The performance gap appears larger for sparse initializations, where active exploration is more important, and becomes smaller as the initialization grid becomes denser. The decrease and convergence of Tr(P) for all methods reflects progressive uncertainty reduction as additional data is collected; however, the informative Dubins– ${\text{RRT}}^{*}$ planner generally reaches lower uncertainty levels more rapidly, suggesting a more efficient reduction of posterior variance over the mission. The improvement with respect to the baseline methods is less visible when compared with ${\text{RRT}}^{*}$ , since the proposed approach differs mainly through the adaptive sampling component, and this difference is reduced as the initialization grid becomes denser and more prior information is available. This suggests that the proposed adaptive sampling strategy helps acquire informative measurements more quickly, although its relative impact becomes attenuated as prior knowledge of the environment increases.

**TABLE 3:** Final average error and Tr(P) across n=5 trials for bathymetric mapping tests.

Planner	Init. Grid size	Mean MAE ±1σ	Mean Tr(P)±1σ
OURS	2	0.0395 ± 0.0030	28.9507 ± 1.8608
MYOP	2	0.0588 ± 0.0055	53.5023 ± 9.1501
GreedyCover	2	0.0742 ± 0.0028	74.6293 ± 5.0396
HEXCover	2	0.0764 ± 0.0093	99.3235 ± 37.7694
${RRT}^{*}$	2	0.0496 ± 0.0071	37.7380 ± 5.6068
OURS	5	0.0339 ± 0.0016	22.4961 ± 0.6873
MYOP	5	0.0447 ± 0.0041	26.0481 ± 4.0898
GreedyCover	5	0.0429 ± 0.0038	34.3665 ± 5.8305
HEXCover	5	0.0470 ± 0.0039	27.9107 ± 5.1156
${RRT}^{*}$	5	0.0360 ± 0.0047	25.9288 ± 2.9725
OURS	10	0.0283 ± 0.0007	18.7103 ± 18.7103
MYOP	10	0.0307 ± 0.0013	20.4167 ± 0.5773
GreedyCover	10	0.0321 ± 0.0013	25.7984 ± 2.7247
HEXCover	10	0.0313 ± 0.0017	24.8593 ± 2.6980
${RRT}^{*}$	10	0.0281 ± 0.0013	19.3116 ± 2.2292

#### 5.5.2 Seagrass mapping extension

Seagrass mapping is appropriately framed as an extension to an obstacle-free approach, as this open-ocean mapping does not include obstacles. This is modeled as a probability density map. Final metric values are summarized in Table 4.

**TABLE 4:** Final average error and Tr(P) across n=5 trials for seagrass mapping tests.

Planner	Init. Grid size	Mean MAE ±1σ	Mean Tr(P)±1σ
OURS	2	0.1012 ± 0.0062	151.5305 ± 21.7870
MYOP	2	0.1407 ± 0.0132	304.3439 ± 42.7580
GreedyCover	2	0.1611 ± 0.0290	418.9714 ± 112.9067
HEXCover	2	0.1672 ± 0.0196	415.2902 ± 35.5770
${RRT}^{*}$	2	0.1066 ± 0.0061	205.1347 ± 17.0459
OURS	5	0.0902 ± 0.0072	91.1672 ± 9.4956
MYOP	5	0.1540 ± 0.0041	124.9973 ± 34.6629
GreedyCover	5	0.1300 ± 0.0121	270.0208 ± 52.5498
HEXCover	5	0.1375 ± 0.0127	216.2761 ± 32.9380
${RRT}^{*}$	5	0.1032 ± 0.0068	113.1466 ± 13.1465
OURS	10	0.0598 ± 0.0031	54.4391 ± 2.3989
MYOP	10	0.0742 ± 0.0071	89.5290 ± 15.1224
GreedyCover	10	0.0687 ± 0.0.0079	104.1760 ± 14.3207
HEXCover	10	0.0816 ± 0.0092	109.8621 ± 13.8067
${RRT}^{*}$	10	0.0619 ± 0.0027	60.9923 ± 7.6388

**FIGURE 15:**
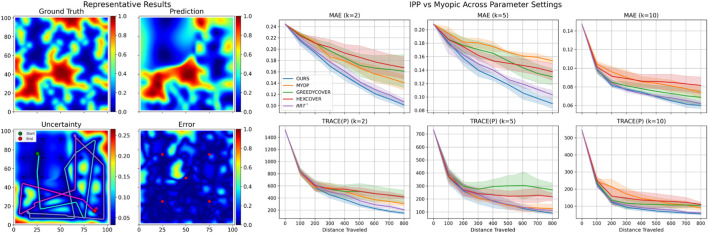
(Left) Representative results for seagrass mapping for a total distance of 800 m and a starting grid of 5×5 , showing (clockwise from top left): the ground truth, the prediction, the absolute error with starting points for all trials, and the uncertainty (σ) overlaid with the robot path for a sample Informative Dubins– ${\text{RRT}}^{*}$ trial. (Right) MAE and Tr(P) results comparing baseline planners and the Informative Dubins– ${\text{RRT}}^{*}$ approach for different initialization settings in the seagrass mapping environment. The evaluated starting grids are 2×2 , 5×5 , and 10×10 . These metrics are shown with ±1σ bounds based on n=5 trials.

As before, the informative Dubins– ${\text{RRT}}^{*}$ planner achieves greater performance in terms of MAE and uncertainty reduction than the baseline methods, including the myopic planner, standard ${\text{RRT}}^{*}$ , GreedyCover, and HEXCover. Based on the final prediction as seen in Figure 15, 100% coverage is not achieved when using a 5×5 initialization grid. This behavior is mainly driven by two factors. First, the initial position and early transects reveal high uncertainty in the upper-right corner and the lower portion of the image, which directs the planner toward those regions. Second, the upper-left corner contains relatively low values and is already partially covered by the initialization grid. Despite the incomplete coverage, the Informative Dubins– ${\text{RRT}}^{*}$ approach still exceeds the performance of the baseline planners across all three initialization grid sizes based on the final mapping results. As before, it is expected that the difference is smaller for the densest initialization grid, as the much denser starting grid reduces the margin of difference between models by exhausting the sampling space. Regardless, across all three parameter values, a greater rate of error reduction is observed for the informative planner relative to the alternative methods. As with the bathymetric mapping case, Tr(P) decreases and converges, with more efficient model uncertainty reduction for the Informative Dubins– ${\text{RRT}}^{*}$ planner.

### 5.6 Stage III: Dubins– ${\text{RRT}}^{*}$ - AK-GPR evaluation (obstacle-rich)

While the previous applications in the obstacle-free section focused on open-ocean applications to justify the absence of obstacles, presented in this section are environments in which boundaries and obstacles are enforced. A natural example of this is marine surveying for algal plumes, which may include coastline or lake shores. Due to the removal of illegal initialization points in the starting grid, a slightly denser minimum starting grid is employed to ensure an adequate number of starting samples, increasing the minimum grid size from 2×2 to 3×3 .

The framework is first evaluated in an environment with synthetic obstacles to introduce additional complexity. After comparing the performance of the baseline planners and the Informative Dubins– ${\text{RRT}}^{*}$ planner, the evaluation is extended to two additional scenarios representing coastal environments, both semi-enclosed and enclosed. Whether synthetic or derived from coastal images, obstacles are generated using binary image masks. The same environment-checking logic is implemented across the planners under comparison to ensure consistency.

#### 5.6.1 Synthetic obstacles and grid density evaluation

**FIGURE 16:**
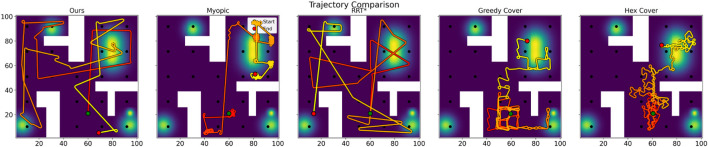
Comparison of trajectories for (left) myopic and (right) Dubins– ${\text{RRT}}^{*}$ approaches in the obstacle-rich synthetic environment for a total run distance of 800 m . The initialization point grid is shown in black. Arrows designate the direction of motion. The trajectories are plotted over the ground truth.

**FIGURE 17:**
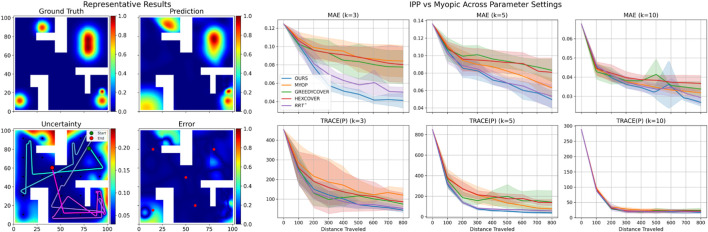
(Left) Representative results for the synthetic obstacles mapping environment with a total distance of 800 m and a starting grid of 5×5 , showing (clockwise from top left): the ground truth, the prediction, the absolute error with starting points for all trials, and the uncertainty (σ) overlaid with the path followed. (Right) MAE and Tr(P) results comparing the baseline planners and the Informative Dubins– ${\text{RRT}}^{*}$ approach for different initialization parameters in the synthetic obstacle-rich environment. Results reflect starting grids of 3×3 , 5×5 , and 10×10 , where points lying on obstacles are excluded from consideration. MAE and Tr(P) metrics are overlaid with ±1σ based on n=5 trials.

The first environment considered in this evaluation stage is synthetic, with obstacles overlaid. These obstacles are shown in Figures 16, 17. Consistent with previous evaluation stages, three different initialization grids are used for evaluation.

The difference in exploration strategies is visible in Figure 16. As in the prior obstacle-free case, the Informative Dubins– ${\text{RRT}}^{*}$ planner achieves a favorable tradeoff between coverage and fine exploration of high-value regions. This behavior remains consistent in the presence of obstacles, where free-space constraints limit exploration. Compared to the myopic and coverage-based planners, the proposed method avoids both excessive local sampling and purely geometric coverage patterns, resulting in more balanced trajectories.

When compared to ${\text{RRT}}^{*}$ , the difference is more subtle; however, the proposed approach exhibits small trajectory adjustments driven by adaptive sampling, leading to more consistent refinement of informative regions rather than the longer, less targeted connections observed in the baseline.

Overall, the results presented in Table 5 show improved mean mapping performance of the Informative Dubins– ${\text{RRT}}^{*}$ planner compared to the baseline planners; however, this performance gap diminishes as the number of points in the initialization grid increases. This behavior reflects the fundamental differences between the strategies. The myopic and coverage-based planners perform better when sufficient coverage is already provided through initialization samples, since less long-range exploration is required. In contrast, the Informative Dubins– ${\text{RRT}}^{*}$ and standard ${\text{RRT}}^{*}$ planners demonstrate stronger performance. In sparse-data scenarios, where trajectory-level optimization and accumulated information gain are more critical, the proposed approach exhibits better performance. In this synthetic obstacle-rich environment, spatial variation is localized to several isolated regions, while large portions of the map exhibit limited variability. Consequently, as initialization density increases, all planners are able to collect enough informative samples for the AK-GPR model to reconstruct the field, leading to convergence in performance metrics such as MAE and Tr(P) . In simpler or more structured environments, the iterative framework allows the AK-GPR model to rapidly reduce uncertainty, causing model adaptation to have a larger influence on the final result and reducing differences between planning strategies.

**TABLE 5:** Final average error and Tr(P) across n=5 trials for the synthetic environment with obstacles.

Planner	Init. Grid size	Mean MAE ±1σ	Mean Tr(P)±1σ
OURS	3	0.0412 ± 0.0084	43.2778 ± 11.9296
MYOP	3	0.0843 ± 0.0173	120.3155 ± 37.3684
GreedyCover	3	0.0775 ± 0.0183	74.5660 ± 12.6625
HEXCover	3	0.0802 ± 0.0194	85.7596 ± 45.6908
${RRT}^{*}$	3	0.0502 ± 0.0056	52.1589 ± 10.2316
OURS	5	0.0495 ± 0.0098	37.2409 ± 6.9386
MYOP	5	0.0634 ± 0.0107	76.6161 ± 14.1740
GreedyCover	5	0.0831 ± 0.0132	141.9154 ± 111.9692
HEXCover	5	0.0808 ± 0.0167	137.6864 ± 30.4994
${RRT}^{*}$	5	0.0534 ± 0.0129	55.3262 ± 16.5912
OURS	10	0.0269 ± 0.0020	17.3074 ± 2.9806
MYOP	10	0.0319 ± 0.0020	22.9276 ± 3.5338
GreedyCover	10	0.0338 ± 0.0038	23.8627 ± 7.0752
HEXCover	10	0.0367 ± 0.0041	21.5004 ± 4.5095
${RRT}^{*}$	10	0.0294 ± 0.0034	19.2927 ± 4.9915

As shown in Figure 17, the proposed framework reproduces most peaks accurately. Specifically, in the scenario with a 5×5 initial grid, most of the corners are already covered during initialization, which shifts the remaining uncertainty toward the right and bottom regions of the map and consequently biases sampling in that direction. The MAE and Tr(P) curves indicate faster convergence for the Informative Dubins– ${\text{RRT}}^{*}$ planner, highlighting the balanced nature of the approach that prioritizes coverage and information gain simultaneously. In the later stages, boosting and penalization become more influential, promoting localized search in uncovered regions. This behavior is reflected in long transects combined with smaller loops that further increase accumulated information gain. Importantly, even when performance differences are reduced, the proposed method remains comparable to sampling-based baselines, indicating that the integration of adaptive sampling does not degrade performance.

#### 5.6.2 Algal mapping extension

Based on the previous results, the planner can be deployed for measuring algal plumes. The purpose of this section is to demonstrate that performance holds with more realistic obstacles. This includes two different boundary types: a non-enclosed boundary and a fully enclosed body of water.

The first test environment uses satellite imagery of a large algal bloom off the coast of Peru. This represents a coastal boundary that does not fully enclose the test space but that introduces the challenge of an irregular boundary.

**FIGURE 18:**
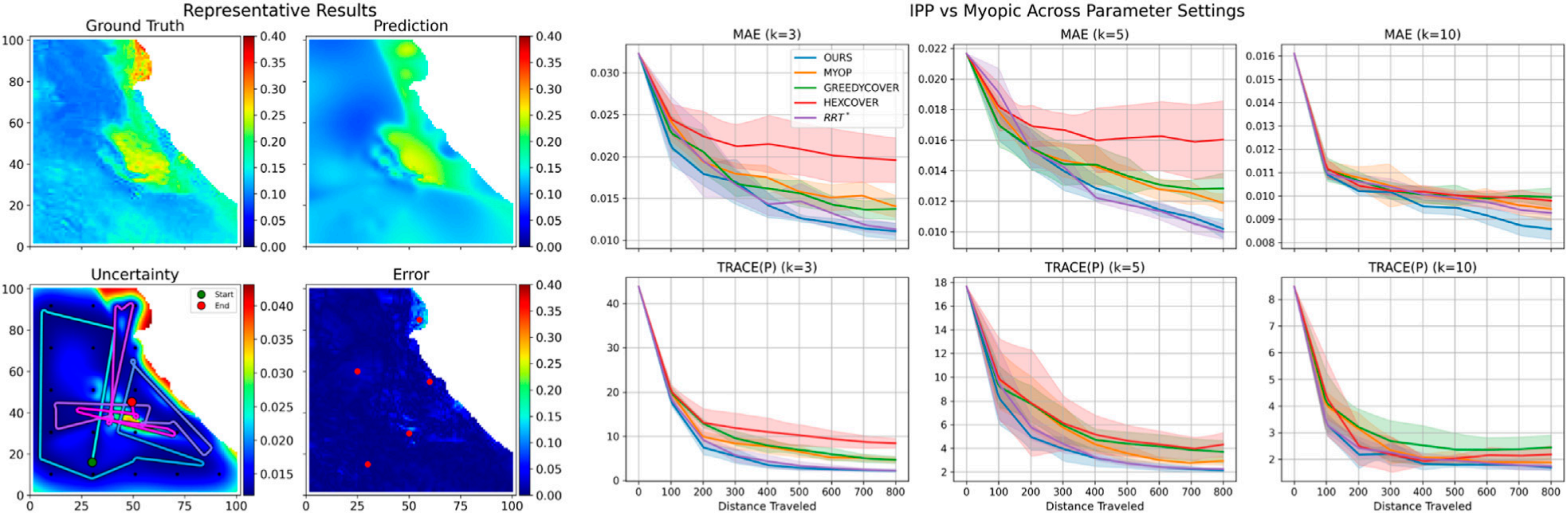
(Left) Representative results for algal mapping with a coastal boundary and a total distance of 800 m and 5×5 starting grid, showing (clockwise from top left): the ground truth, the prediction, error (absolute) with starting points for all trials, and uncertainty (σ) overlaid with robot path. (Right) MAE and Tr(P) results comparing the baseline planners and the Informative Dubins– ${\text{RRT}}^{*}$ approach, corresponding to different initialization parameters in the coastal boundary mapping environment. Results reflect a starting grid of 3×3 , 5×5 , and 10×10 , where points lying on obstacles are excluded from consideration. These metrics are overlaid with ±1σ based on n=5 trials.

The results for the coastal planning environment are consistent with the previous synthetic obstacle case. In the representative trial shown in Figure 18, the main algal patches, including the higher concentration region located near the coast, are reproduced by the Informative Dubins– ${\text{RRT}}^{*}$ planner. The mean MAE converges more quickly for the Informative Dubins– ${\text{RRT}}^{*}$ planner than for the baseline methods, although the performance of ${\text{RRT}}^{*}$ in this scenario is close. In general, the gap between all methods becomes smaller as the number of starting samples increases. A similar trend is observed for the Tr(P) values. As shown in Table 6, the results begin to overlap for the 10×10 grid case. Tradeoffs between broad coverage and local refinement are visible in the trajectory, with the same combination of longer transects and focused loops observed in the synthetic obstacle experiments. As in previous environments, the distinction between planners is greater for sparser grids, such as the 3×3 and 5×5 initializations. This supports the use of the Informative Dubins– ${\text{RRT}}^{*}$ planner in unexplored environments and its ability to explore globally and locally rather than purely locally. The Peru environment exhibits lower overall spatial variability, with informative regions concentrated near the coastline. As a result, the mapping task is easier for all planners, which reduces the performance gap between methods. This is also reflected in the lower range of MAE and Tr(P) values compared with the previous environments.

**TABLE 6:** Final average error and Tr(P) across n=5 trials for coastal algae mapping tests.

Planner	Init. Grid size	Mean MAE ±1σ	Mean Tr(P)±1σ
OURS	3	0.0111 ± 0.0010	2.2158 ± 0.3237
MYOP	3	0.0141 ± 0.0013	4.6526 ± 0.6069
GreedyCover	3	0.0137 ± 0.0013	4.6590 ± 0.7999
HEXCover	3	0.0196 ± 0.0027	8.4418 ± 1.0738
${RRT}^{*}$	3	1.0738 ± 0.0007	2.2623 ± 0.1877
OURS	5	0.0102 ± 0.0006	2.1425 ± 0.0853
MYOP	5	0.0119 ± 0.0006	2.9370 ± 0.3662
GreedyCover	5	0.0128 ± 0.0010	3.7089 ± 0.9378
HEXCover	5	0.0160 ± 0.0025	4.3227 ± 1.0111
${RRT}^{*}$	5	0.0100 ± 0.0005	2.2421 ± 0.1115
OURS	10	0.0086 ± 0.0004	1.6826 ± 0.1035
MYOP	10	0.0094 ± 0.0005	1.8709 ± 0.1620
GreedyCover	10	0.0099 ± 0.0004	2.4426 ± 0.4600
HEXCover	10	0.0098 ± 0.0003	2.1810 ± 0.3244
${RRT}^{*}$	10	0.0093 ± 0.0001	1.7325 ± 0.1403

The final test environment for evaluating algorithm performance is a fully enclosed environment. This is drawn from Baltic Sea aerial imagery. This environment exhibits a relatively short range of values across the map. However, unlike the Peru environment, it does not contain large regions with uniform values, and instead shows more localized spatial variability. Despite having the smallest free-space area ( ∼ 53%), the spatial variability is distributed across the environment, making the mapping task more complicated than the Peru environment.

**FIGURE 19:**
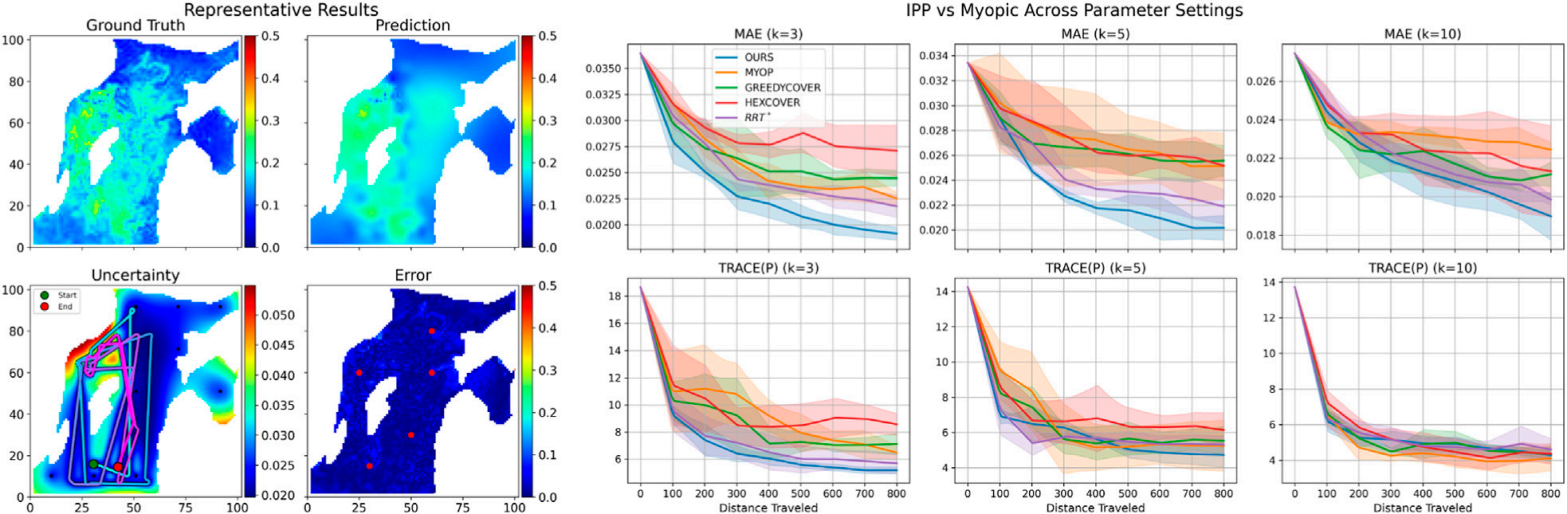
(Left) Representative results for algal mapping in an enclosed environment for a total distance of 800 m , showing (clockwise from top left): the ground truth, the prediction, error (absolute) with starting points for all trials, and uncertainty (σ) overlaid with robot path. (Right) MAE and Tr(P) results comparing the baseline planners and the Informative Dubins– ${\text{RRT}}^{*}$ approach, corresponding to different initialization parameters in the coastal boundary mapping environment. Results reflect a starting grid of 3×3 , 5×5 , and 10×10 , where points lying on obstacles are excluded from consideration. These metrics are overlaid with ±1σ based on n=5 trials.

The results for this environment, summarized in Table 7, are similar to those seen for the other obstacle-rich environments. As before, the Informative Dubins– ${\text{RRT}}^{*}$ planner achieves a lower mean MAE than the baseline planners. From the sample trajectory shown in Figure 19, the proposed approach explores locally at a greater rate than in previous environments, as evidenced by the loop artifacts near the high-value regions. Despite this behavior, coverage is not sacrificed because the Informative Dubins– ${\text{RRT}}^{*}$ planner also forces exploration, resulting in greater mapping performance. Opposed to the behavior observed in previous environments, denser grids do not necessarily yield better mapping performance, mainly due to the reduced area and the presence of localized high-value regions, which reduce the need to explore a large portion of the map. As a result, performance is comparable across initialization grid sizes.

**TABLE 7:** Final average error and Tr(P) across n=5 trials for enclosed algae mapping tests.

Planner	Init. Grid size	Mean MAE ±1σ	Mean Tr(P)±1σ
OURS	3	0.0192 ± 0.0006	5.1836 ± 0.2561
MYOP	3	0.0225 ± 0.0006	6.4773 ± 0.5103
GreedyCover	3	0.0245 ± 0.0007	7.1209 ± 0.7773
HEXCover	3	0.0271 ± 0.0024	8.5753 ± 0.7893
${RRT}^{*}$	3	0.0218 ± 0.0011	5.7007 ± 0.7595
OURS	5	0.0202 ± 0.0010	4.7218 ± 0.7095
MYOP	5	0.0251 ± 0.0027	5.2475 ± 1.4386
GreedyCover	5	0.0256 ± 0.0012	5.5350 ± 0.8059
HEXCover	5	0.0252 ± 0.0012	6.1361 ± 0.9827
${RRT}^{*}$	5	0.0219 ± 0.0014	5.3006 ± 0.5051
OURS	10	0.0190 ± 0.0012	4.2770 ± 0.2753
MYOP	10	0.0224 ± 0.0004	4.1030 ± 0.7068
GreedyCover	10	0.0212 ± 0.0006	4.3409 ± 0.4175
HEXCover	10	0.0213 ± 0.0024	4.3555 ± 0.5755
${RRT}^{*}$	10	0.0198 ± 0.0003	4.5970 ± 0.6248

Uncertainty reduction results are less distinct than in prior environments, with the Informative Dubins– ${\text{RRT}}^{*}$ planner outperforming the baseline planners primarily for the 3×3 initialization grid. For the 5×5 and 10×10 grids, all methods converge to similar final Tr(P) values, with only minor differences between planners. This behavior may be explained by two factors. First, the Baltic environment contains a relatively small free-space area, requiring less exploration to achieve proper coverage. Since the iteration budget is fixed across the environments, each transect covers a larger portion of the environment. Second, this dense coverage favors localized uncertainty resolution, allowing greedy or local planners to efficiently reduce uncertainty by sampling nearby regions. Consequently, all planners achieve similar overall Tr(P) values. Although several methods achieve comparable uncertainty reduction in these cases, this does not necessarily translate into equivalent mapping performance. Baseline planners exhibit either locally focused uncertainty reduction or explicit coverage-driven behavior, whereas the Informative Dubins– ${\text{RRT}}^{*}$ planner balances local refinement with broader environmental coverage. This produces more spatially diverse observations and improves global reconstruction accuracy, as reflected in the lower MAE values. This highlights the importance of balancing uncertainty reduction with spatial coverage in informative sampling.

## 6 Conclusions and future work

We have presented a closed-loop uncertainty-guided informative path planning framework based on Dubins– ${\text{RRT}}^{*}$ and adaptive sampling under budget constraints for ASVs intended for ocean exploration and ecological monitoring tasks. The proposed algorithm was methodically evaluated through several stages. First, an ablation study showed that adaptive sampling enables faster convergence to improved solutions when compared with RRT and ${\text{RRT}}^{*}$ . In addition, a second study helped clarify the role of AK-GPR within the overall framework, showing that more frequent retraining events can reduce the relative influence of the planner on the final result. Subsequently, the performance of the AK-GPR model was assessed using the WAM-V 16 ASV platform with a myopic-style planner. Although good mapping performance was achieved, larger deviations were observed between trials, likely induced by the constant looping behavior of the local planner. These results motivated the development of a planning strategy using the validated AK-GPR model, combined with a planner that explicitly accounts for vehicle kinematic constraints while improving global exploration.

The framework was subsequently evaluated in five representative environments relevant to ecological and ocean monitoring applications. In all environments, the proposed IPP approach showed improved average mapping performance, particularly in scenarios with sparse initial data. This was demonstrated through consistent reductions in both MAE and Tr(P) , with an average improvement of approximately 24% in MAE and 32% in predictive uncertainty compared to baseline planners. The average improvement was more pronounced in the sparse initialization scenarios, reaching 33% in MAE and 42% in reduction of uncertainty. Conversely, the average improvement was smaller for denser initialization grids, with approximately 15% improvement in MAE and 23% in uncertainty reduction. Additionally, obstacle avoidance was validated in environments representative of coastal monitoring scenarios, such as tracking algal plumes. In obstacle-rich environments, the performance gap between planners was smaller, primarily due to the lower spatial variability present in these maps. Nevertheless, the IPP framework maintained improved performance, particularly in the sparse initialization cases, where global exploration is most critical. These results highlight the importance of trajectory-based informative planning strategies that balance uncertainty reduction with spatial coverage, allowing more efficient environmental exploration.

While the proposed framework is validated across diverse simulated environments and supported by field verification of the modeling component, full real-world deployment of the closed-loop informative planner remains an important direction for future work. Additionally, some baseline methods were adapted to align with the continuous-space and Dubins-constrained formulation, which may affect direct comparability. Finally, the evaluation focuses on static environments; extending the framework to dynamic or time-varying fields is a key next step.

Future work includes expanding the test scenarios explored here to a more robust simulation framework in preparation for field deployment. This includes the incorporation of other sensors with distinct behavior from the point sensor. Further work is suggested in studying other uses for non-stationary kernels and iterating on the GP aspect of this work. Finally, future iterations of this work may explore the use of multiple sensors for environmental mapping, including collaborative mapping across multiple robotic platforms, and incorporating these capabilities into the AK-GPR model.

The work presented here is a foundation for the continued development of IPP technologies onboard robotic platforms, and the proposed future work will help develop it further to improve environmental mapping technologies.

## Data Availability

The raw data supporting the conclusions of this article will be made available by the authors, without undue reservation.
